# Diversity and complexity of cell death: a historical review

**DOI:** 10.1038/s12276-023-01078-x

**Published:** 2023-08-23

**Authors:** Wonyoung Park, Shibo Wei, Bo-Sung Kim, Bosung Kim, Sung-Jin Bae, Young Chan Chae, Dongryeol Ryu, Ki-Tae Ha

**Affiliations:** 1 Department of Korean Medical Science, School of Korean Medicine, Pusan National University, Yangsan, Gyeongsangnam-do 50612 Republic of Korea; 2 Korean Medical Research Center for Healthy Aging, Pusan National University, Yangsan, Gyeongsangnam-do 50612 Republic of Korea; 3 Department of Precision Medicine, School of Medicine, Sungkyunkwan University School of Medicine, Suwon, Gyeonggi-do 16419 Republic of Korea; 4 Department of Molecular Biology and Immunology, Kosin University College of Medicine, Busan, 49267 Republic of Korea; 5 Department of Biological Sciences, UNIST, Ulsan, 44919 Republic of Korea; 6 Department of Biomedical Science and Engineering, Gwangju Institute of Science and Technology, Gwangju, 61005 Republic of Korea

**Keywords:** Apoptosis, Autophagy, Entosis, Necroptosis

## Abstract

Death is the inevitable fate of all living organisms, whether at the individual or cellular level. For a long time, cell death was believed to be an undesirable but unavoidable final outcome of nonfunctioning cells, as inflammation was inevitably triggered in response to damage. However, experimental evidence accumulated over the past few decades has revealed different types of cell death that are genetically programmed to eliminate unnecessary or severely damaged cells that may damage surrounding tissues. Several types of cell death, including apoptosis, necrosis, autophagic cell death, and lysosomal cell death, which are classified as programmed cell death, and pyroptosis, necroptosis, and NETosis, which are classified as inflammatory cell death, have been described over the years. Recently, several novel forms of cell death, namely, mitoptosis, paraptosis, immunogenic cell death, entosis, methuosis, parthanatos, ferroptosis, autosis, alkaliptosis, oxeiptosis, cuproptosis, and erebosis, have been discovered and advanced our understanding of cell death and its complexity. In this review, we provide a historical overview of the discovery and characterization of different forms of cell death and highlight their diversity and complexity. We also briefly discuss the regulatory mechanisms underlying each type of cell death and the implications of cell death in various physiological and pathological contexts. This review provides a comprehensive understanding of different mechanisms of cell death that can be leveraged to develop novel therapeutic strategies for various diseases.

## Cell death mechanisms: implications for disease therapies

Cell death is a complex and interconnected process that plays a crucial role in maintaining tissue homeostasis and preventing disease. There are various types of cell death, including necrosis, apoptosis, autophagy, and others, each with distinct morphological features and molecular mechanisms. Understanding the diverse processes underlying cell death is essential for understanding diseases and developing new therapies. Recent research has focused on characterizing and distinguishing various forms of cell death, thereby advancing our understanding of their roles in health and disease. The complex mechanisms underlying cell death are underscored by the intricate interconnections among different types of cell death and the regulation of these mechanisms through diverse signaling pathways and environmental factors. Further research is necessary to fully characterize and differentiate among the various forms of cell death and their roles in pathological conditions.

## Introduction

Cell death is a biological process that results in the cessation of cell function and, eventually, cell death^1^. Its main function is to maintain tissue homeostasis by removing nonfunctional, damaged, and harmful cells^2^. Although this is a natural process involved in tissue formation, maintenance, and repair, it can also be triggered in response to injury, disease, or damage, leading to pathological cell death^3–5^.

Until the 19th century, death was understood only at the individual organism level, and the concept of cell death was not readily accepted by physicians and biologists. After the development of light microscopy, tissue sectioning practices, and staining techniques, death at the cellular level was recognized^6,7^. Despite cellular theory advocates such as botanist Mathias Jakob Schleiden and zoologist Theodore Schwann in the 1830s–1860s, the use of microscopes in medicine was limited. The application of technical advances in light microscopy and the concept that organisms are composed of cells to medicine was led by the German pathologist Rudolf Virchow^6^. In his papers published in 1855^8^ and a book, “Cellular Pathology,” based on a compilation of lectures he gave in 1858^9^, Virchow formalized cellular pathology as the fundamental basis of pathology. This new perspective radically changed the way pathology was viewed^1,6^. Virchow first recognized necrosis as death at the cellular level during his study of cellular changes accompanying tissue damage caused by inflammation^6,10^.

In 1842, German anatomist Carl Vogt first proposed that spontaneous cell death was a physiological phenomenon. He reported that cell death during metamorphosis in the midwife toad eliminated the notochord and allowed vertebrae to develop^7^. In 1882, the Russian biologist Elie Metchnikoff, considered a pioneer in modern immunology, observed that phagocytic cells engulfed dying cells in several organisms^11^. Metchnikoff’s discovery meaningfully contributed to our understanding of the role of the immune system in eliminating dying cells and maintaining tissue homeostasis. Later, in 1972, pathologist John F. Kerr and his colleagues discovered a kind of cell death that differed from necrosis, which they named “apoptosis”^12,13^. The discovery of apoptosis was a fundamental hallmark in the study of cell death and expanded our understanding of various types of cell death.

**Fig. 1:**
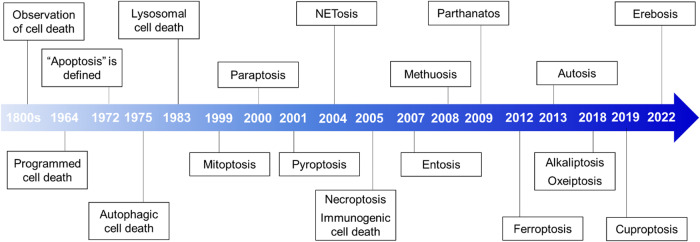
Timeline of the discovery of cell death. This timeline depicts the important discoveries and advancements in cell death research, including the recognition of multiple forms of cell death.

Traditional classifications of cell death include necrosis and programmed cell death (PCD). Necrosis, a nonprogrammed form of cell death, is often caused by traumatic injury; PCD, a controlled form of cell death, results from a series of molecular events in response to various physiological or developmental signals^14^. Apoptosis is a well-characterized PCD mechanism^15^. Other types of PCD, including autophagic cell death^16^, lysosomal cell death^17^, mitoptosis^18^, paraptosis^19^, pyroptosis^20^, NETosis^21^, necroptosis^22^, immunogenic cell death^23^, entosis^24^, methuosis^25^, parthanatos^26^, ferroptosis^27^, autosis^28^, alkaliptosis^29^, oxeiptosis^30^, cuproptosis^31^, and erebosis^32^, have also been identified (Fig. 1; Table 1). To date, the study of cell death is a major field of research in biology^2^. Morphological features are the primary basis for the traditional classification of cell death. In 2018, the Nomenclature Committee on Cell Death (NCCD) published comprehensive information, which expanded our knowledge of cell death pathways, and the assays commonly used in cell death study^2^. It emphasized the importance of accurately characterizing and differentiating different types of cell death and highlighted the importance of molecular pathways, genetic factors, biochemical markers, and functional criteria. With increasing understanding of the complexity of cell death, this classification system became more complicated and now includes additional categories. Understanding the various types of cell death and their regulatory mechanisms is essential for evaluating the pathogenesis of various illnesses, such as cancer, neurodegenerative diseases, and autoimmune disorders^4,5^.

**Table 1:** Characteristics of the major types of cell death.

Classification	Type	Morphological features	Key regulator molecules	Major inhibitors (target)
Non-RCD	Necrosis	Cell swelling, plasma membrane rupture, loss of organelle	-	-
RCD-apoptotic	Apoptosis (Anoikis)	Cell shrinkage, nuclear condensation, apoptotic body fragmentation, nuclear membrane rupture, plasma membrane blebbing, loss of positional organization of organelles	Death receptors and their ligands, Bax, Bak, Bcl-2, AIF, caspase-3, caspase-8, caspase-9, TP53	Z-VAD-FMK, emricasan, Q-VD-OPh, Z-VAD(OH)-FMK (pancaspase), Z-DEVD-FMK (caspase-3, -6, -7, and -10), Z-VDVAD-FMK (caspase-2), ivachtin (caspase-3), Ac-DEVD-CHO (caspase-3 and -7), Z-IETD-FMK (caspase-8), Q-LEHD-OPh (caspase-9)
RCD-vacuole presenting	Autophagic cell death (Autosis)	Autophagic vacuolization, plasma membrane blebbing, organelle enlargement, depletion of cytoplasmic organelles	AMPK, mTOR, ULK1, PI3KIII, BECN1, ATGs, LC3, Na^+^/K^+^-ATPase	Chloroquine (lysosome), bafilomycin A1, concanamycin A (H^+^-ATPase), 3-methyladenine, wortmannin (PI3K), spautin 1(USP10 and USP13)
	Entosis	Cell-in-cell structure	RhoA, ROCKI/II, E-cadherin, α-catenin, actomyosin, LC3, ATGs	C3-toxin (RhoA), Y-27632 (ROCK), blebbistatin (myosin)
	Methuosis	Accumulation of large fluid‑filled single-membrane vacuoles, cell swelling, plasma membrane rupture	Ras, Rac1, Arf6, LAMP1, Rab7	SP600125 (JNK)
	Paraptosis	Accumulation of large fluid‑filled single-membrane vacuoles, dilation of the ER or mitochondria	mHCX, mNCX, MCU, VDAC, PyR, IP_3_R, SERCA, MAPKs	Actinomycin D, cycloheximide (ER stress)
RCD-mitochondria dependent	Mitoptosis	Mitochondria disappearance, decomposition of the mitochondrial reticulum into small spherical organelles	Bax, Bak, TIMM8a (DPP), Drp1	NAC, trolox (ROS)
	Parthanatos	Chromatic condensation, large DNA fragment formation, lack of apoptotic bodies and small-scale DNA fragments, loss of membrane integrity, lack of cell swelling	PARP, AIF	BYK204165, AG-14361, iniparib (PARP1)
RCD-metal dependent	Ferroptosis	Small mitochondria, reduced number of mitochondrial cristae, elevated mitochondrial membrane density, increased rate of mitochondrial membrane rupture	System X_C_^-^, GPX4, lipid ROS	Deferoxamine, ciclopirox, deferiprone (Fe), ferrostatin-1, liproxstatin-1, β-mercaptoethanol, vitamin E, β-carotene, NAC, XJB-5-131, zileuton, CoQ10, baicalein (ROS), vildagliptin, alogliptin, linagliptin (DPP4), thiazolidinedione, rosiglitazone (ACSL4), selenium (GPX4)
	Cuproptosis	Mitochondrial shrinkage	SLC31A1, ATP7B, FDX1	GSH (chelate Cu), UK5099 (MPC), rotenone, antimycin A (ETC)
RCD-immunogenic	Necroptosis	Cell swelling, plasma membrane rupture, moderate chromatin condensation	Death receptors, TLRs, TCR, RIPKs, MLMK	Tetrahydroisoquinolines, lactoferrin, DNase (NETs), cl-amidine (PADI4)
	Lysosomal cell death	Lysosomal and plasma membrane rupture	Cathepsins, STAT3, TP53, NF-κB, and MCOLN1	CA-074Me (CTSB), deferoxamine (Fe), NAC (ROS)
	Pyroptosis	Lack of cell swelling, plasma membrane rupture, bubbling, moderate chromatin condensation and fragmentation	NLRs, ALRs, caspase-1, caspase-11, GSDMD	Disulfiram, LDC7559, Ac-FLTD-CMK, Polyphyllin VI (GSDMD), morroniside (MMP2/9)
	NETosis	Plasma membrane rupture, nuclear membrane collapse, chromatic fiber release	NOX4, PADI4	Tetrahydroisoquinolines (NETs), cl-amidine (PADI4)
Other	Alkaliptosis	Necroptosis-like morphology	NF-κB, IKBKB, CA9	NAC, *N*-acetyl alanine acid (pH), IMD0354, CAY10657, SC514 (IKBKB)
	Oxeiptosis	Apoptosis-like morphology	KEAP1, PGAM5, AIFM1	NAC (ROS)
	Erebosis	Loss of proteins, organelles, and nuclear contents; ACE accumulation; shortened microvilli; fragmented nuclei	Unknown	Unknown

*RCD* regulated cell death, *ACE* angiotensin-converting enzyme, *GSDMD* gasdermin D.

In this historical review, we provide an overview of different types of cell death and the morphological and biochemical characteristics of cells undergoing these types of cell death. We also discuss the current understanding of the molecular mechanisms underlying the different types of cell death and the significance of these mechanisms in normal physiology and disease. We hope that this review serves as an excellent resource for researchers investigating cell death.

## Discovery of diverse types of cell death

### Necrosis

Necrosis is an uncontrolled form of cell death that is triggered in response to injury, trauma, or infection^33^. The term “necrosis” comes from the Greek words “*nekros*,” meaning dead or corpse, and “*osis*,” meaning process, referring specifically to destruction and degeneration^34^. It is unclear who initially coined the term necrosis, but the earliest reference to necrosis recently found on PubMed is “Observation on Necrosis” written by Bouffelin, a surgeon in the Polish army, in 1786^35^. In the literature, necrosis refers to tissue necrosis caused by infection and inflammation, not cell death. As mentioned in the Introduction, Rudolf Virchow was the first scientist to use the term necrosis at the cellular level. He defined necrosis at the cellular level as “the mortified cell is left in its external form” and “necrobiosis or shrinkage necrosis being where the cell vanishes and can no longer be seen in its present form”^6,10^. In 1877, Carl Weigert and Julius Konheim described certain lesions as exhibiting coagulative necrosis, a basic form of necrosis, and necrobiosis^1,6^.

**Fig. 2:**
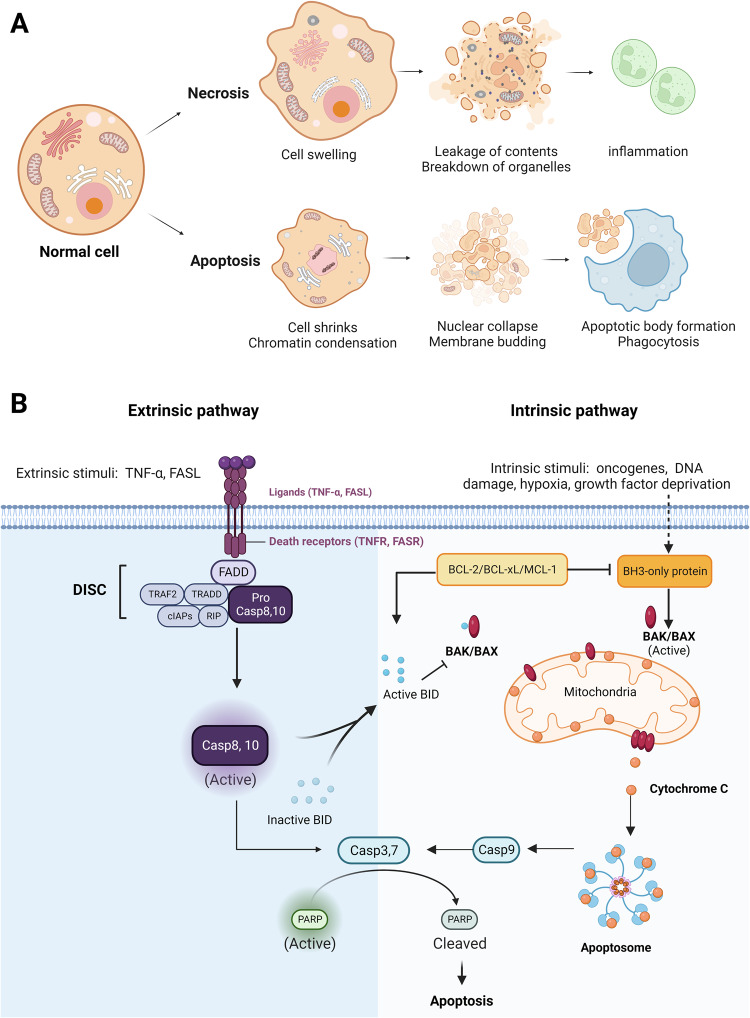
Necrosis and apoptosis: morphological features and signaling pathways. **A** Hallmarks of necrosis and apoptosis are illustrated. Necrosis is an uncontrolled and pathological form of cell death, marked by cell swelling, membrane rupture, and intracellular content release, leading to inflammation and tissue damage. In contrast, apoptosis is a tightly controlled form of cell death that involves characteristic morphological features, such as cell shrinkage, chromatin condensation, membrane blebbing, nuclear fragmentation, and apoptotic body formation. **B** The two signaling pathways that lead to apoptosis are described. The extrinsic pathway is initiated by the binding of death ligands, such as tumor necrosis factor (TNF)-α or Fas ligand (FasL), to death receptors, which activates caspase 8. The intrinsic pathway, regulated by the Bcl-2 family, is triggered by intracellular stressors, such as DNA damage and oxidative stress, resulting in the release of cytochrome c from mitochondria and activation of caspase 9. The two pathways ultimately converge on caspase 3, which mediates the execution of apoptosis.

The cellular mechanisms that lead to necrosis are complex and not yet fully understood; however, they generally involve a series of events that result in the breakdown of cellular components and the release of cell contents into the extracellular space (Fig. 2A)^36^. In contrast to apoptosis, necrosis is frequently associated with inflammation and damage to surrounding tissues because the intracellular contents released by dying cells can activate the immune system and harm neighboring cells^37^.

### Apoptosis

Apoptosis is characterized by the organized breakdown of cells in response to particular signals^38^. The term “apoptosis” comes from the Greek words “*apo*,” meaning leaf, and “*ptosis*,” meaning “falling off,” which describe the process of cells undergoing controlled self-destruction and detachment from the surrounding tissue^12^. The apoptotic process was first described by Carl Vogt in 1842, and it was rediscovered and named “apoptosis” by Kerr in 1972^39^. As mentioned in the Introduction, Carl Vogt was the first to present the concept of spontaneous cell death as a physiological phenomenon^7^. In 1885, Walther Flemming described and illustrated a cellular process called chromatolysis, which he discovered while studying the degeneration of ovarian follicles; Franz Nissen, who observed pigment degradation in lactating mammary glands, published similar results^1,6^. In 1914, Ludwig Gräffer published a paper proposing the premise that some mechanism must balance mitosis and that this process likely involves the process Fleming described as chromatolysis; this paper was subsequently rediscovered by Alfred Glücksmann in 1951^1^. Subsequently, experimental pathologists began to investigate more intensively how cells in different organs die during development and in response to stimuli or injuries.

We now know that spontaneous cell death, such as chromatolysis, is caused by apoptosis. The description of apoptosis as a distinct form of cell death that differs from necrosis was formalized in the early 1970s by Australian pathologist John Kerr and his colleagues^6,39^. As a graduate student in London in 1962, Kerr found that when the portal venous blood supply to the liver is cut off, a different type of death occurs in cells around distal hepatic veins. This form of cell death was characterized by cytoplasmic shrinkage and condensed nuclear chromatin fragments, in addition to certain manifestations of classical necrosis. Upon further investigation using histochemical and electron microscopy, the newly discovered mode of cell death appeared to be nondegenerative in nature and was called shrinkage necrosis^40^. In 1972, Kerr was on sabbatical at the University of Aberdeen in Scotland working with Professor Currie and graduate student Andrew Wiley, who realized that a newly characterized type of cell death was regulated by hormones and played an essential role in normal development. Recognizing the inappropriateness of using the term necrosis for cell death under physiological conditions, they proposed that this process be called apoptosis^41,42^.

Apoptosis is characterized by cell shrinkage, chromatin condensation, and fragmentation into small membrane-bound apoptotic bodies, which are phagocytosed by adjacent parenchymal cells, neoplastic cells, or macrophages^15^ (Fig. 2A). Apoptosis is a genetically regulated and controlled cell death process, whereas necrosis is an uncontrolled cell death process caused by external stimuli. Apoptosis leads to cell fragmentation and removal by phagocytic cells, whereas necrosis resulted in cell membrane rupture and inflammation^43^ (Fig. 2A).

Two main pathways lead to the activation of apoptosis: the extrinsic and intrinsic pathways^44^. The extrinsic pathway is activated by extracellular ligands (tumor necrosis factor [TNF]-α and Fas ligand [FasL]) binding to death receptors, namely, TNF and Fas receptors, respectively^45,46^. After extracellular ligands bind to death receptors, death-inducing signaling complexes (DISCs) are formed and recruit and activate initiator caspases, such as caspase-8 and caspase-10^47^. These initiator caspases then cleave and activate effector caspases, such as caspase-3, -6, and -7, leading to the degradation of intracellular components and the induction of apoptosis^48^. The intrinsic pathway is activated by intracellular stressors, such as DNA damage, oxidative stress, and loss of survival signaling, which lead to permeabilization of the outer mitochondrial membrane^49^. This pathway is regulated by the Bcl-2 family of antiapoptotic proteins (Bcl-2 and Bcl-xL), proapoptotic proteins (Bax and Bak), and BH3-only proteins (Bim and Bid)^50^. In response to intracellular stress, the activation of proapoptotic BH3-only proteins inhibits antiapoptotic proteins, allowing Bax and Bak to form mitochondrial pores and release cytochrome c into the cytosol^51^. The released cytochrome c forms an apoptosome with apoptotic protease activating factor-1 (Apaf-1) and activates caspase-9 (Fig. 2B)^52^.

### Autophagic cell death

Autophagic cell death, also known as type 2 cell death, occurs as a result of the activation of the autophagy pathway^53^. Autophagy is a cellular process in which cytoplasmic components, including organelles and macromolecules, are sequestered in double-membrane autophagosomes and targeted for lysosomal degradation^54^. The term “autophagy” comes from the Greek words “*auto*,” meaning self and “*phagy*,” meaning eating or devouring, and describes the process by which cells degrade and recycle their components^55^. The term “autophagy” has been used since the mid-19th century, but Christian de Duve defined the word as it is currently used in 1963 based on his research on lysosomal functions^56^. The mechanisms underlying autophagy were deduced in the 1990s with the identification of autophagy-related genes by Yoshinori Ohsumi^57^. Autophagic cell death was first described by Timo J. Nevalainen in 1975^58^.

Under normal conditions, autophagy helps maintain cell homeostasis and recycle nutrients while removing toxic cellular components^59^. However, under certain conditions, such as nutrient deprivation, oxidative stress, or exposure to cytotoxic agents, autophagy can become dysregulated and result in cell death^60^.

**Fig. 3:**
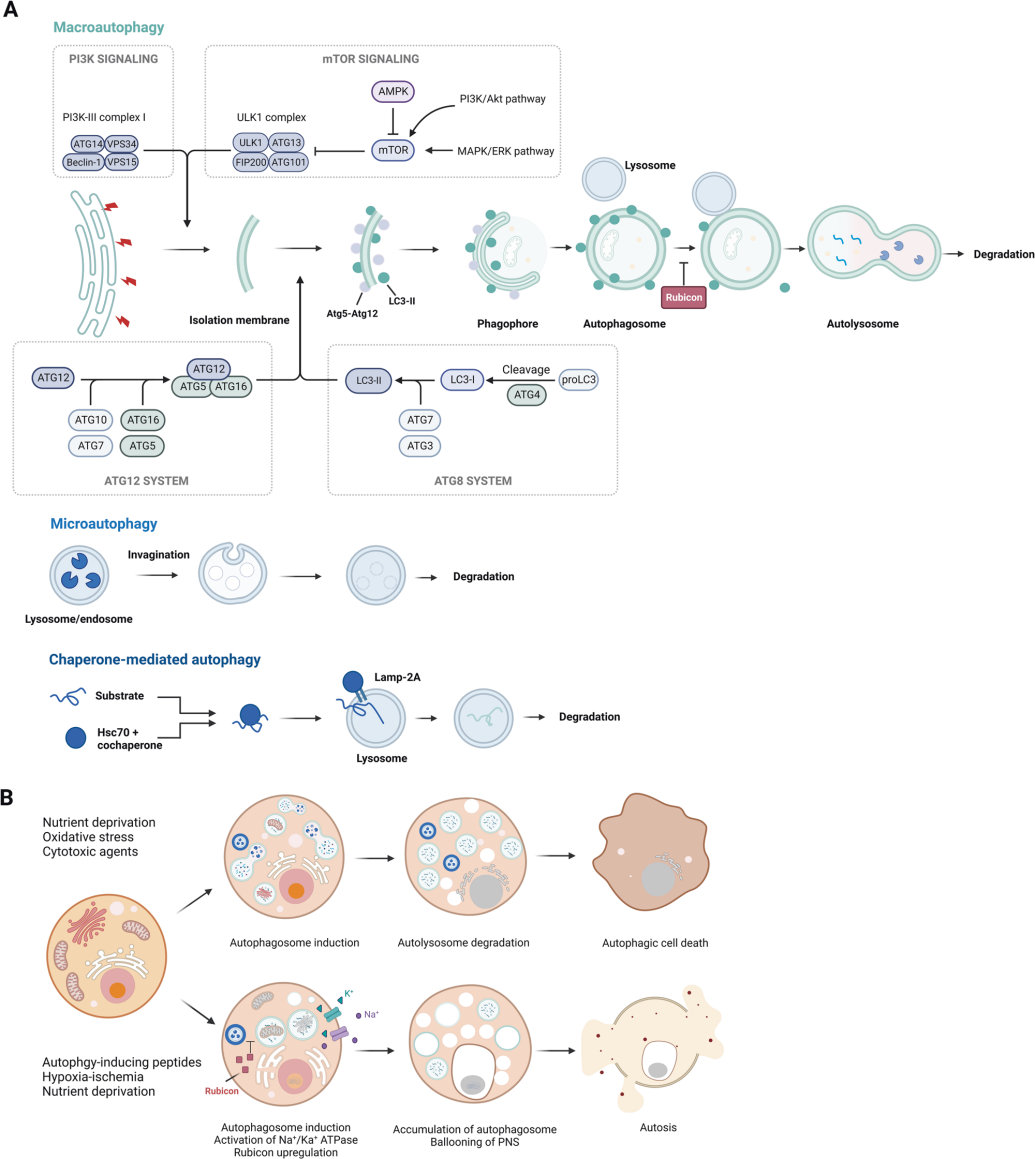
Progression and morphological features of autophagy-mediated cell death. **A** The figure shows three types of autophagy, namely, macroautophagy, microautophagy, and chaperone-mediated autophagy. Macroautophagy involves the formation of double-membrane vesicles that engulf cytoplasmic components and organelles and then fuse with lysosomes to form autolysosomes. Microautophagy involves engulfing cytoplasmic components and organelles directly into lysosomes. Chaperone-mediated autophagy degrades specific proteins via chaperone proteins that transport them to lysosomes. **B** The figure highlights the contrast between autophagy and autosis, two processes involving autophagy. While autophagic cell death is a result of excessive autophagy, autosis is characterized by three distinct phases characterized by cells with unique morphological features and is triggered by various signals, such as Na^+^/K^+^-ATPase, Tat-Beclin 1, and hypoxia–ischemia.

The first step in the activation of autophagy is the formation of an isolation membrane, or phagophore, which is a double-membrane structure that sequesters cytoplasmic components to be degraded^61^. Phagophore formation requires the action of the unc-51-like kinase 1 (ULK1) complex, which is composed of several proteins, namely, ULK1, ATG13, FIP200/RB1CC1, and ATG101^62^. The ULK1 complex is regulated by several signaling pathways, including the mTOR pathway. Under normal conditions, the activation of mTOR suppresses autophagy by inhibiting the formation of the ULK1 complex; however, under stress or nutrient-deprivation conditions, mTOR inhibition leads to the formation of the ULK1 complex and the initiation of autophagy^63^. The phagophore expands and sequesters the cytoplasmic components to be degraded and then fuses with lysosomes to form autolysosomes, where the sequestered contents are degraded by lysosomal enzymes^64^. Autophagy is regulated by a complex network of proteins, including the ATG family of proteins, Beclin-1, and microtubule-associated protein light chain 3 (LC3)^65^. ATG proteins are involved in various steps in the autophagy pathway, including phagophore formation, elongation, and closure^66^. The Beclin-1 protein is required for the formation of the phagophore, whereas the LC3 protein is involved in the elongation and closure of the phagophore and the maturation of the autophagosome (Fig. 3A)^61^. Autophagy activation initially leads to the development of large cytoplasmic vacuoles, which in turn cause autophagic cell death^16^. Autophagy promotes both cell survival and cell death^67^. Notably, in some cases, autophagy contributes to cancer cell resistance to chemotherapy^68^. However, in other cases, autophagy can induce cell death and inhibit tumor growth^69^.

According to the NCCD, autophagic cell death is a regulated form of cell death that relies on autophagy machinery and can be prevented only by blocking autophagy^2^. Autophagic cell death has been implicated in various physiological and pathological processes, including cancer, neurodegeneration, ischemic injury, and heart disease^70–74^. However, the precise role of autophagy in cell death is still unclear^16^. Therefore, further research is necessary to clarify the role of autophagy in cell death. This research may have important implications for the understanding and treatment of various diseases.

### Autosis

Autosis is an autophagy-dependent type of cell death that was discovered in 2013, and its name is derived from the Greek words “*autos*,” meaning self, and “*osis*,” meaning a process or condition^75^. Autosis is characterized by unique cell morphology and depends on cellular Na^+^/K^+^-ATPase activity^75^. Autosis is triggered by various signals, such as cerebral hypoxia–ischemia, nutrient deprivation, and autophagy-inducing peptides (Tat-Beclin)^75^.

The morphological features of autosis are acquired in three distinct phases: phase 1a, marked by a dilated and fragmented endoplasmic reticulum (ER) and an increase in the number of autophagosomes, autolysosomes, and empty vacuoles; phase 1b, involving swelling of the perinuclear space (PNS) in the presence of cytoplasmic materials and electron-dense mitochondria; and phase 2, marked by a reduced number of cytoplasmic organelles, focal nuclear concavity, and PNS ballooning (Fig. 3B)^76^. Autosis is triggered in vitro in Tat-Beclin 1-treated cells and in vivo in the brains of neonatal rats undergoing challenged with hypoxia–ischemia^75^. Rubicon levels significantly increase during autosis, which prevents the fusion of autophagosomes with lysosomes and inhibits autophagosome maturation and degradation^77,78^. Autosis is likely caused by the excessive accumulation of autophagosomes, which can deplete intracellular organelle membranes, such as ER and mitochondrial membranes, leading to reduced organelle function and, in the case of mitochondria, depolarization and loss of mitochondrial membrane potential^79,80^. Cardiac glycosides that inhibit the Na^+^/K^+^-ATPase pump can prevent autosis and contribute to the treatment of heart injuries^75^. However, the molecular mechanisms underlying the regulation of autosis by Na^+^/K^+^-ATPases remain unclear.

A recent study revealed that myxoma virus can infect and proliferate in human tumor cells but not in normal cells and that infected chimeric antigen receptor (CAR)-T and T-cell receptor (TCR)-T cells can efficiently trigger autotic cell death both in vitro and in vivo^81^. Autosis has also been observed in patients with severe liver diseases, including acute liver insufficiency associated with severe anorexia nervosa^82^. Although much remains unknown about autosis, its discovery has opened new avenues of research into the complex and diverse mechanisms underlying PCD.

### Lysosomal cell death

Lysosomal cell death results from lysosomal membrane permeabilization, which causes the release of lysosomal enzymes into the cytoplasm and activation of cell death pathways^83^. The concept of lysosomal cell death was first proposed by Christian de Duve in the late 1990s^84^. Lysosomes are organelles that contain hydrolases critical for degrading intracellular and extracellular material. Under normal physiological conditions, lysosomes play roles in maintaining cellular homeostasis^85,86^. However, after the lysosomal membrane is damaged, lysosomal hydrolases are released into the cytoplasm, triggering various cell death pathways^87^.

**Fig. 4:**
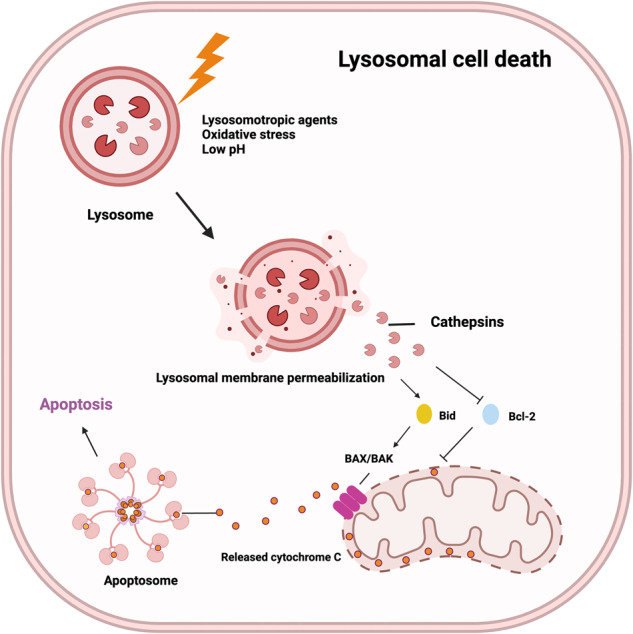
Mechanism of lysosomal cell death. This figure illustrates lysosomal cell death caused by lysosomal membrane permeabilization and the release of lysosomal enzymes into the cytoplasm, leading to the activation of apoptotic cell death pathways. Lysosomal cell death can be induced by stimuli, such as changes in lysosomal pH, oxidative stress, and lysosomotropic agents. The release of lysosomal proteases, such as cathepsins, activates the lysosomal apoptotic pathway by cleaving Bid and degrading antiapoptotic Bcl-2 homologs.

Lysosomal cell death can be induced by various stimuli, including changes in lysosomal pH, oxidative stress, and lysosomotropic agents^88^. Lysosomal proteases, such as cathepsins, have been identified as potential causes of lysosomal cell death because they can be released into the cytoplasm and activate the lysosomal apoptotic pathway by cleaving Bid and degrading antiapoptotic Bcl-2 homologs following lysosomal injury and targeted destabilization of the lysosomal membrane (Fig. 4)^89,90^.

Lysosomal cell death is thought to be involved in various pathological conditions, such as neurodegenerative diseases, cancer, and age-related disorders, and the inhibition of lysosomal cell death may hold therapeutic potential for these diseases^91^. Further research is necessary to fully understand the mechanisms underlying lysosomal cell death and its role in pathological conditions.

### Mitoptosis

Mitoptosis, also known as mitochondrial suicide, is a PCD that involves dysfunctional mitochondria and was first proposed by Vladimir P. Skulachev in 1999^92^. Dysfunctional mitochondria are associated with numerous diseases, including cancer, neurodegenerative disorders, and metabolic diseases^93^. Mitoptosis and mitophagy (autophagic degradation of mitochondria) are crucial for preventing the accumulation of dysfunctional mitochondria, which can lead to various cellular pathologies^94^.

**Fig. 5:**
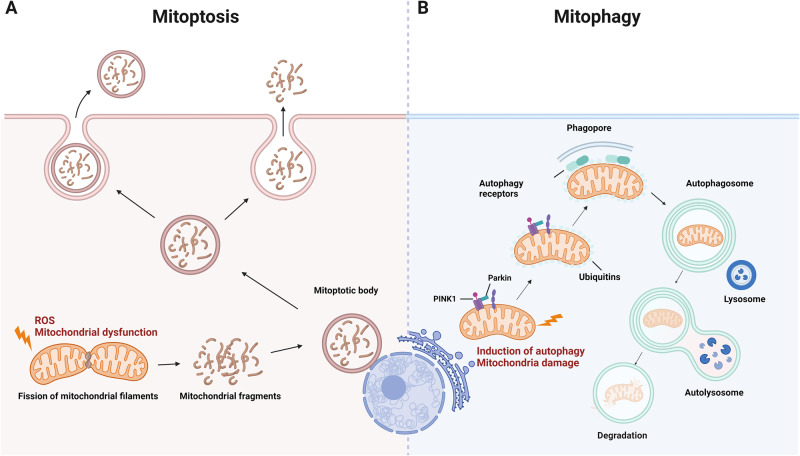
Comparison of mitoptosis and mitophagy. This figure illustrates the crucial processes of mitoptosis and mitophagy that maintain mitochondrial quality and prevent cell pathology. **A** Mitoptosis is characterized by several events, including mitochondrial fission, the clustering of spherical mitochondria in the perinuclear area, enwrapping of these clusters by a membrane to form a “mitoptotic body,” decomposition of mitochondria into small vesicles, protrusion of the body from the cell, and disruption of the boundary membrane. This process is driven by mitochondrial dysfunction and reactive oxygen species (ROS) production. **B** Mitophagy is a selective autophagic mechanism for the degradation of damaged or unnecessary mitochondria. This procedure requires activation of general autophagy and priming of injured mitochondria via the Pink1/Parkin signaling pathway. Autophagosomes engulf targeted mitochondria, which are then digested and degraded in lysosomes.

Mitoptosis is triggered by mitochondrial dysfunction and reactive oxygen species (ROS) production^95^. Mitoptosis is involved in several biological processes, such as cell differentiation, hematopoietic stem cell self-renewal, metabolic remodeling, and elimination of paternal mitochondria in organisms for which mitochondrial DNA is maternally inherited^96^. The steps involved in mitoptosis include the fission of mitochondrial filaments to form spherical mitochondria, clustering of these spherical mitochondria in the perinuclear area, occlusion of these mitochondrial clusters via a membrane that forms a "mitoptotic body," decomposition of mitochondria inside this body into small membrane vesicles, protrusion of the body from the cell, and finally, disruption of the boundary membrane (Fig. 5A)^95^. Notably, autophagy is not involved in the mitoptotic process. Different forms of mitoptosis have been observed, including inner- and outer-membrane mitoptosis^18^. In inner membrane mitoptosis, only the internal matrix and cristae of mitochondria are degraded, and the external mitochondrial envelope remains unaltered^18^. During outer membrane mitoptosis, the internal cristae of mitochondria swell and undergo fragmentation, and the outer mitochondrial membrane bursts, releasing the remnants of cristae into the cytoplasm^18^.

In contrast to mitoptosis, mitophagy selectively degrades damaged mitochondria through autophagy, which requires the induction of general autophagy and priming of damaged mitochondria mediated by the Pink1/Parkin signaling pathway (Fig. 5B)^97^. The main difference between mitoptosis and mitophagy is that mitoptosis targets dysfunctional mitochondria, which are subsequently degraded inside the “mitoptotic body,” leading to membrane disruption. In contrast, mitophagy selectively degrades damaged or otherwise undesirable mitochondria. Although both processes are important for maintaining cellular homeostasis by eliminating dysfunctional or excessive mitochondria, their mechanisms of action differ.

Mitoptosis is important for the elimination of damaged or dysfunctional mitochondria and the maintenance of cellular homeostasis^18^. Recent research on mitoptosis has been focused on understanding the mechanisms that regulate this process and developing strategies for targeting dysfunctional mitochondria in disease contexts.

### Immunogenic cell death

Immunogenic cell death (ICD) is a type of PCD in which an immune response is triggered by the release of damage-associated molecular patterns (DAMPs) from dying cells, which attract immune cells to the site of cell death^98^. The ICD concept was first proposed by the group led by Guido Kroemer and Laurence Zitvogel in 2005^99^.

**Fig. 6:**
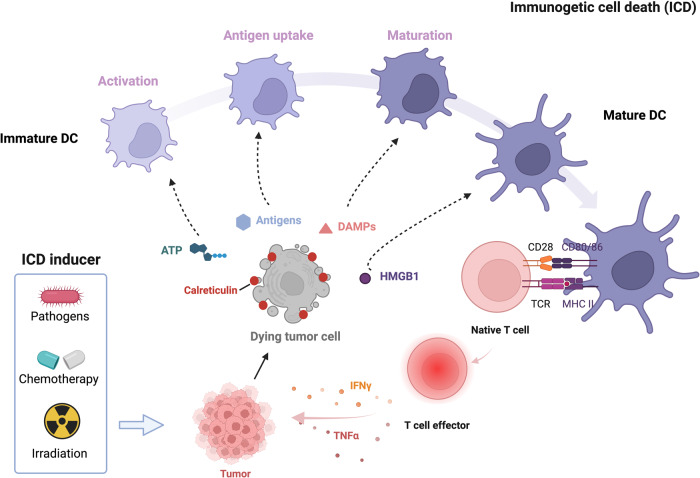
Mechanism underlying immunogenic cell death (ICD). This figure illustrates the mechanism of ICD and its potential as a cancer therapeutic strategy. During ICD, dying cells release damage-associated molecular patterns (DAMPs), such as ATP, high-mobility group box 1 (HMGB1), and heat shock proteins (HSPs), which activate dendritic cells (DCs) and other immune cells, promoting antigen presentation and immune activation. Effector T cells release interferon (IFN)-γ and TNFα, which activate other immune cells, such as natural killer cells and macrophages that detect and eliminate cancer cells.

During ICD, dying tumor cells express calreticulin on their surface, which functions as an “eat me” signal to dendritic cells (DCs) and other phagocytic cells^100^. This signaling promotes phagocytosis of the dying cells by the DCs, leading to the activation of an immune response. ICD also involves the release of DAMPs, such as ATP, high-mobility group box 1, and heat shock proteins (HSPs), from dying cells^101^. These DAMPs activate DCs and other immune cells, thereby promoting antigen presentation and immune activation^102^. Moreover, IFNγ and TNFα released by effector T cells attract and activate other immune cells, including natural killer cells and macrophages, which detect and eradicate cancer cells (Fig. 6)^103^.

ICD has emerged as a promising strategy for cancer therapy. It potentially enhances the effectiveness of cancer treatments, such as chemotherapy and radiotherapy, which in turn induce ICD in cancer cells^104^. ICD-based therapies provide long-lasting protection against cancer recurrence and metastasis by promoting immune responses against cancer cells.

### Pyroptosis

Pyroptosis is a type of PCD that involves inflammation and is mediated by caspase-1^105^. It was first discovered by Brad Cookson and Molly Brennan in 2001; it is a novel form of caspase-1-dependent PCD in immune cells, such as macrophages and dendritic cells, that defends the body against intracellular pathogens^106^. However, in contrast to other forms of PCD, pyroptosis contributes to tissue damage in inflammatory disorders^107^.

**Fig. 7:**
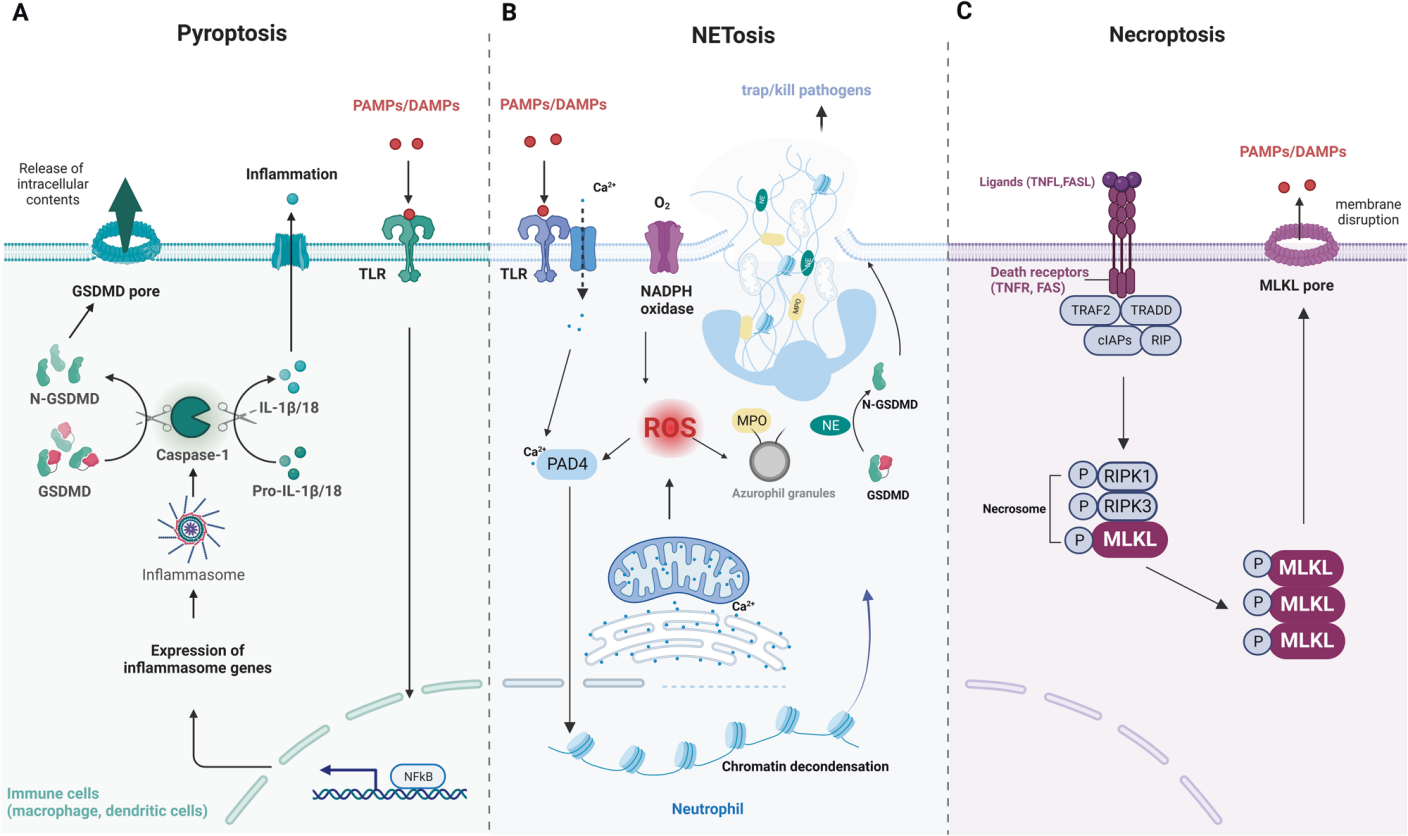
Mechanisms of pyroptosis, NETosis, and necroptosis. **A** Pyroptosis is characterized by cell swelling, plasma membrane rupture, and the release of proinflammatory cytokines, such as interleukin (IL)-1β and IL-18. Pyroptosis is triggered by the activation of inflammasomes, cytoplasmic complexes that sense danger signals, and initiate a caspase-1-dependent cascade that ultimately leads to cell death. **B** NETosis is a process in which neutrophils release DNA fibers coated with antimicrobial peptides to trap and kill pathogens. During NETosis, neutrophils undergo marked morphological changes, including chromatin decondensation, nuclear envelope rupture, and granule mixing, leading to the formation of neutrophil extracellular traps (NETs). The release of NETs is triggered by various stimuli, such as pathogens, cytokines, and immune complexes. **C** Necroptosis is mediated by death receptors. Upon activation of death receptors, such as TNFR1, receptor-interacting protein kinase 1 (RIPK1) binds to RIPK3 to form a necrosome. The necrosome complex promotes the oligomerization and phosphorylation of the mixed lineage kinase domain-like protein (MLKL). The oligomeric form of MLKL is translocated from the cytosol to the plasma membrane, leading to the formation of membrane pores and subsequent plasma membrane rupture. This results in the release of damage-associated molecular patterns (DAMPs), which trigger inflammation.

Pyroptosis is initiated by the activation of pattern recognition receptors (PRRs) in response to pathogen-associated molecular patterns (PAMPs) or DAMPs, which trigger inflammasome assembly^108^. The inflammasome is a protein complex consisting of PRRs, adaptor proteins, and caspase-1, with caspase-1 cleaving gasdermin D (GSDMD) to produce an N-terminal GSDMD fragment that forms membrane pores^109^. Caspase-1 also activates the proinflammatory interleukins, interleukin-1 beta (IL-1β) and interleukin-18 (IL-18) activity, also via proteolysis^110^. The actions of caspases result in the release of proinflammatory cytokines and the recruitment of immune cells to the site of infection or injury (Fig. 7A)^111^.

Pyroptosis has been implicated in several pathological conditions, including infectious diseases, autoimmune disorders, cancer, and neurodegenerative diseases^112,113^. Studies have shown that inhibition of pyroptosis can alleviate inflammation and tissue damage in the contexts of these conditions^114,115^. Therefore, targeting pyroptosis may be a potential therapeutic strategy for the treatment of inflammatory diseases.

### NETosis

NETosis is a type of PCD characterized by the release of neutrophil extracellular traps (NETs) into the extracellular space^116^. NETs are web-like structures composed of chromatin, histones, and granular proteins that are released by neutrophils, a type of white blood cell, to capture and kill invading pathogens, including bacteria, viruses, and fungi^117^. NETosis was first described by Volker Brinkmann et al. in 2004^118^.

The mechanism of NETosis activation involves a series of complex molecular events, including ROS production, nuclear envelope disassembly, chromatin decondensation, and NET release (Fig. 7b)^116^. One of the key events in NETosis is the activation of the NADPH oxidase complex, which depends on an increase in the cytoplasmic concentration of Ca^2+^ and subsequent ROS production^116^. When ROS are activated, protein complexes known as “azurosomes” dissociates from azurophil granules dissociates and causes NE, cathepsin G, azurocidin, and MPO to be released into the cytosol, where they contribute to chromatin decondensation and nuclear envelope disintegration^119^. Another important factor in NETosis is peptidyl-arginine deaminase 4 (PAD4), which is transferred from the cytoplasm to the nucleus to catalyze the citrullination of histones, leading to chromatin decondensation^120^. Histones can also undergo acetylation during NETosis; however, the role of this process is not clearly understood^121^. In the final stage of NETosis, pores are formed in the plasma membrane, and chromatin is released into the extracellular environment, and NETs are formed. GSDMD plays a critical role in the formation of these membrane pores. In contrast to pyroptosis, in which GSDMD is activated through caspase-induced cleavage, NETosis is activated mainly by NE^122,123^. In addition, both the ER and mitochondria play important roles in NETosis. NETosis is initiated by calcium release from the neutrophil ER, which triggers the assembly of the NADPH oxidase complex and the generation of ROS^124–126^. Mitochondrial ROS production also promotes NETosis, potentially by regulating NADPH oxidase activity^127^. Overall, the coordinated activation of multiple pathways and organelles is required for successful NETosis.

NETosis plays an important role in the innate immune response because it allows neutrophils to directly combat pathogens^128^. However, excessive or inappropriate NETosis can contribute to the development of inflammatory and autoimmune diseases, such as sepsis, rheumatoid arthritis, lupus, and cancer^129,130^. Therefore, regulation of NETosis is a topic of ongoing research in the field of immunology.

### Necroptosis

Necroptosis is a form of PCD that differs from necrosis and apoptosis in terms of morphology and biochemistry^131^. Necroptosis was first identified and described by Dr. Francis Chan et al. in 2005^132^. It is mediated by a signaling cascade involving the activation of receptor-interacting protein kinase 1 (RIPK1) and RIPK3 and the formation of a complex called the necrosome^133^.

TNFα and TNFR1 ligation triggers a well-characterized necroptosis-inducing pathway^134^. Under normal conditions, stress signals activate caspase-8, leading to the initiation of apoptosis^135^. However, when caspase-8 activity is suppressed, RIPK1 and RIPK3 are activated, leading to necroptosis^136^. During TNF-induced necroptosis, RIPK1 can recruit RIPK3 through the RIP homotypic interaction motif (RHIM) to form necrosomes, which promote the oligomerization and phosphorylation of mixed lineage kinase domain-like protein (MLKL)^137^. The oligomeric form of MLKL is translocated from the cytosol to the plasma membrane, leading to the formation of membrane pores and the subsequent rupture of the plasma membrane, resulting in the release of DAMPs^138^. The released DAMPs are recognized by PRRs on immune cells, leading to the activation of inflammatory responses (Fig. 7c)^108^. This inflammatory response can contribute to the clearance of dead cells and the initiation of tissue repair processes^139^. However, excessive or prolonged inflammation can cause tissue damage and contribute to the pathogenesis of various diseases^140^.

Research results suggest that necroptosis is involved in the pathogenesis of several diseases, including neurodegenerative diseases, viral infections, ischemic injury, and cancer^141–144^. The inhibition of necroptosis has shown therapeutic potential in some disease models, making it an attractive target for drug development^145^.

### Cuproptosis

Cuproptosis is a form of PCD triggered by copper (Cu)^146^. The term “cuproptosis” is derived from the Latin word “*cuprum*,” which means copper, and the Greek word “ptosis,” meaning falling off. This was first described and the term was initially defined by Tsvetkov, P. et al. in 2019 (Nat Chem Biol 15, 681–689, 2019)^147^. Cuproptosis differs from other types of oxidative stress-related cell death, such as apoptosis and ferroptosis, and is characterized by mitochondrial stress caused by the aggregation of lipoylated mitochondrial enzymes and the loss of Fe–S cluster proteins^31^.

**Fig. 8:**
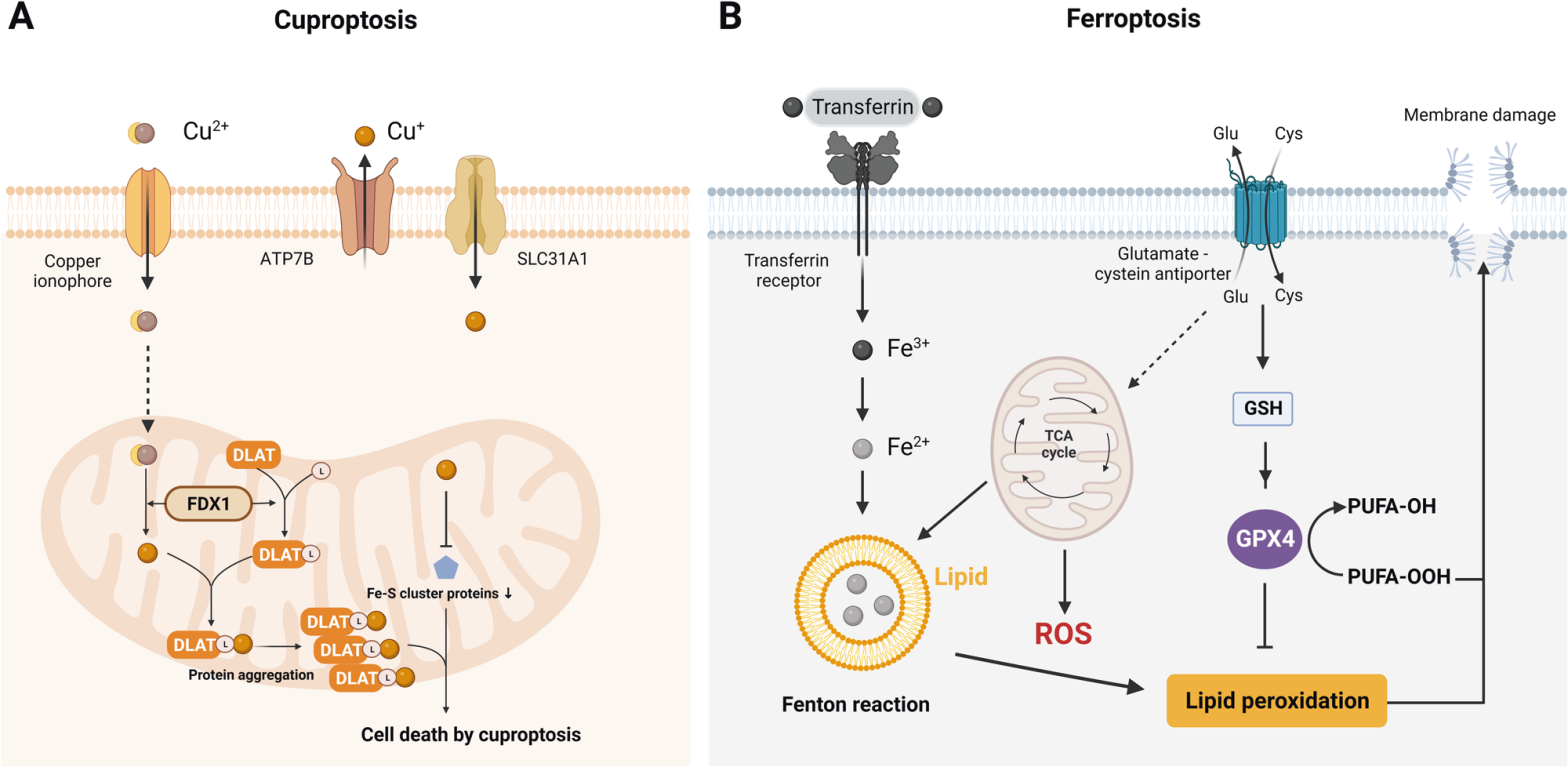
Copper and iron-driven cell death: cuproptosis and ferroptosis. **A** Cuproptosis is triggered by the accumulation of copper. It results in mitochondrial stress due to the aggregation of lipoylated mitochondrial enzymes and the loss of Fe–S cluster proteins, which can be mediated by ferredoxin 1 (FDX1). **B** Ferroptosis is characterized by the depletion of intracellular glutathione and decreased activity of glutathione peroxidase 4 (GPX4), which leads to the accumulation of unmetabolized lipid peroxides and increased ROS production. Membrane damage is also a result of lipid peroxidation.

Copper is an essential trace element that plays vital roles in various biological processes, including oxygen transport, energy production, and antioxidant defense^148,149^. However, excess copper can be toxic to cells and tissues, leading to a condition known as copper overload or copper toxicity^150^. Two mitochondrial proteotoxic stress pathways mediate cuproptosis. The mitochondrial matrix reductase ferredoxin 1 (FDX1) catalyzes the reduction of ES–Cu^2+^ to Cu^+^, releasing it into mitochondria^151^. FDX1 has also been identified as a novel effector of lipoylation that contributes to the accumulation of toxic lipoylated dihydrolipoamide S-acetyltransferase (DLAT)^152^. Cu^+^ binds to lipoylated DLAT, promoting the disulfide bond-dependent aggregation of lipoylated DLAT, which leads to the accumulation of toxic lipoylated DLAT and subsequent cuproptotic cell death^153^. In addition, FDX1-dependent degradation of Fe-S cluster proteins may favor cuproptosis (Fig. 8A)^31^. This type of cell death depends on the amount of copper in cells and the lipoylation status of tricarboxylic acid (TCA) cycle enzymes.

Cuproptosis has been implicated in several pathological conditions, such as Wilson’s disease, a genetic disorder characterized by the accumulation of copper in the liver, brain, and other organs^154^. Therefore, understanding the mechanisms underlying coproptosis may provide insights into the pathogenesis and treatment of copper-related diseases.

### Ferroptosis

Ferroptosis is an iron-dependent form of PCD that involves the accumulation of lipid peroxides and oxidative stress leading to membrane damage^155^. Ferroptosis was first described in 2012 by a research team led by Brent Stockwell^155^. The name ferroptosis comes from the Latin word “*ferrum*,” meaning iron, and the Greek word “*ptosis*,” meaning falling, which together refer to the iron-dependent process of cellular demise^27^. Ferroptosis is thought to play a role in various physiological processes, including ischemia, cancer, and neurodegeneration^156^.

Ferroptosis is regulated by several factors, such as iron metabolism, lipid peroxidation, and antioxidant systems^157^. Ferroptosis is initiated by the accumulation of lipid peroxides generated through the oxidation of polyunsaturated fatty acids via lipoxygenases or other enzymes^158^. Lipid peroxides accumulate through the oxidation of polyunsaturated fatty acids, a process that can be further amplified via the iron-catalyzed Fenton reaction, generating ROS and hydroxyl radicals that attack and damage cellular components, particularly the cell membrane, resulting in cell death (Fig. 8B)^159^. The cystine/glutamate antiporter system imports cystine, a precursor of the antioxidant glutathione, and thus plays a significant role in regulating the accumulation of lipid peroxides and iron^160^. Glutathione neutralizes free radicals and ROS and protects cells from oxidative stress and lipid peroxidation^161^. Ferroptosis is characterized by the depletion of intracellular glutathione and decreased activity of glutathione peroxidase 4 (GPX4), leading to the accumulation of unmetabolized lipid peroxides and the production of high levels of ROS^27,162^. Other factors that can regulate ferroptosis include iron metabolism; the activity of lipid metabolism enzymes, such as acyl-CoA synthetase long-chain family member 4 (ACSL4); and the expression of genes involved in cell stress response pathways, such as the p53 pathway^163,164^. In cancer treatment, inhibition of the cystine/glutamate antiporter system induces ferroptosis^165^. In addition, the use of ferroptosis-inducing agents, such as erastin and RSL3, may become a novel approach to cancer therapy^166^.

Ferroptosis is a unique and important form of PCD with broad implications in various physiological processes and disease states. Further studies may provide new insights into the mechanisms underlying this form of cell death and potential therapeutic interventions.

### Paraptosis

Paraptosis, the name of which is derives from the combination of “*para*”, meaning next to or related to, and “apoptosis,” is a type of PCD that was initially discovered by Sabina Sperandio et al. in 2000^167^. Paraptosis and apoptosis are typically induced simultaneously in cells. In contrast to apoptosis, paraptosis does not involve caspase activation or DNA fragmentation^167^. Paraptosis is characterized by the swelling and vacuolization of the ER and mitochondria, resulting in the formation of large cytoplasmic vacuoles^168^.

**Fig. 9:**
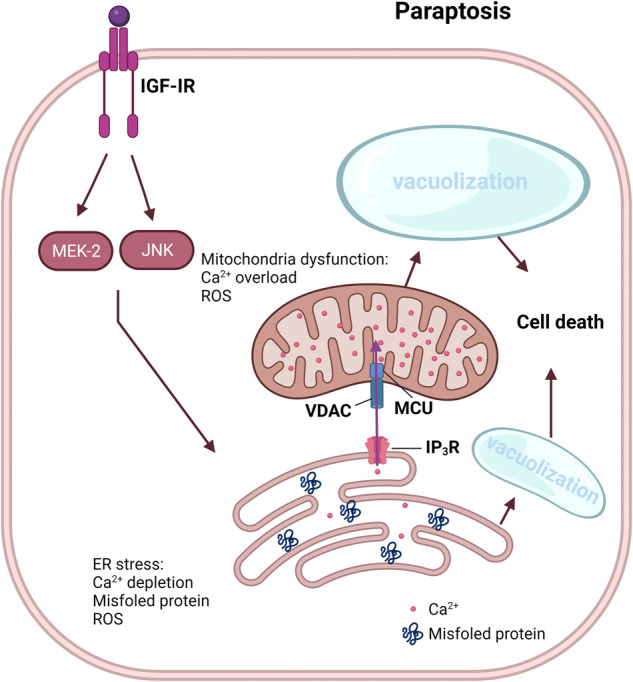
Mechanism underlying paraptosis. Paraptosis is characterized by the development of large vacuoles in the endoplasmic reticulum (ER) and mitochondria, ultimately leading to the formation of large cytoplasmic vacuoles. Impaired proteostasis, altered ion homeostasis, and ER stress cause paraptosis, resulting in the discharge of Ca^2+^ from the ER and accumulation of Ca^2+^ in mitochondria. Paraptosis can be facilitated by the activation of mitogen-activated protein kinase (MAPK) signaling pathways via IGF-IR and inhibited by AIP-1/Alix.

Multiple mechanisms can trigger paraptosis. Impaired proteostasis due to proteasomal inhibition or altered protein thiol homeostasis, as well as unbalanced ion homeostasis, can lead to paraptosis^169^. Paraptosis is characterized by cytoplasmic vacuolization resulting from swelling of the ER and mitochondria. The accumulation of misfolded proteins within the ER lumen leads to the development of an osmotic force that causes water to be drawn away from the cytoplasm, causing ER distension (Fig. 9)^170^. ER stress and dilation can contribute to the release of Ca^2+^ from the ER, which can cause mitochondrial Ca^2+^ overload via an intracellular Ca2+ flux mechanism located at the ER-mitochondrial axis and thus mitochondrial dilatation^169^. Stimulation of the MEK-2 and JNK pathways by IGF-IR, as well as its inhibition mediated by AIP-1/Alix, is known to promote paraptosis^171^. Paraptosis is believed to play a role in various physiological and pathological processes, including embryonic development, neurodegeneration, and cancerogenesis^172^. In cancer cells, paraptosis is induced by various chemotherapeutic agents, including the proteasome inhibitor bortezomib and histone deacetylase (HDAC) inhibitor suberoylanilide hydroxamic acid (SAHA)^173,174^.

In summary, paraptosis is a type of PCD characterized by the formation of large cytoplasmic vacuoles and activation of multiple signaling pathways. Further research is required to fully elucidate the mechanisms underlying paraptosis and its potential as a therapeutic target for various diseases.

### Methuosis

Methuosis is a nonapoptotic form of cell death characterized by the accumulation of vacuoles derived from macropinosomes, which are large endocytic vesicles^175^. The term methuosis is derived from the Greek word for “*methuo*,” meaning to drink to intoxication, and refers to the fact that the vacuoles in methuotic cells appear to be filled with an unknown substance^25^. Methuosis was first described by Overmeyer et al. in 2008^176^.

**Fig. 10:**
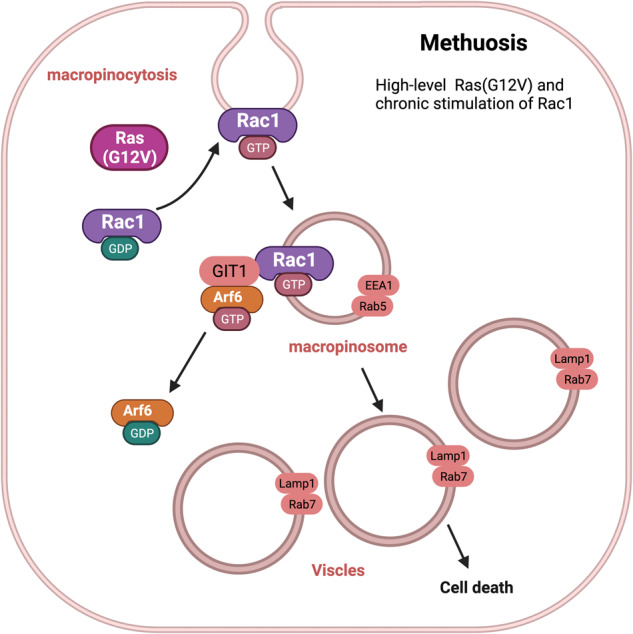
Molecular basis of methuosis. This image depicts the working model of methuosis. Methuosis is initiated by prolonged high-level expression of RAS (G12V) and chronic activation of Rac1, which leads to enhanced macropinocytic activity. Moreover, this mechanism hampers macropinosome recycling by lowering the active Arf6 pool. Nascent macropinosomes, which are created from lamellipodial membrane projections, penetrate the cell and merge to form large fluid-filled vacuoles that, in contrast to typical macropinosomes, cannot be recycled. These vacuoles grow rapidly, resulting in a stable population with certain late endosomal features (Rab7 and LAMP1).

Methuosis is triggered by sustained high-level expression of the activated form of Ras (G12V) and chronic stimulation of Rac1^177^. This stimulation increases the rate of macropinocytic, which is the process of molecules uptake into the extracellular fluid through the formation of large vesicles called macropinosomes^178^. However, methuosis impairs macropinosome recycling by decreasing the pool of active Arf6, which is a protein involved in vesicle trafficking^25^. Thus, macropinosomes accumulate and fuse to form large vacuoles that displace the nucleus and other organelles in a cell. Eventually, the vacuoles become sufficiently large to rupture the cell membrane, leading to cell death (Fig. 10)^179^. However, the exact molecular mechanisms underlying this form of cell death are not fully understood.

Methuosis has been observed in various cancer cell lines and has been proposed to be a potential therapeutic target for cancer treatment^180,181^. However, further research is required to fully understand the molecular mechanisms underlying methuosis and the potential of these mechanisms as targets for cancer therapy.

### Entosis

Entosis is a nonapoptotic form of cell death in which one living cell actively internalizes and degrades another living cell^175^. Entosis is derived from the Greek word “*entos*,” meaning inside or within, and was first described by Overholtzer et al. in 2007^182^. The internalization of a living cell by another cells leads to the formation of a double-membrane vesicle called the entotic vacuole^183^. During normal development, entosis is thought to play a role in the removal of excess cells and in shaping tissues and organs^184^. In cancer, entosis has been shown to contribute to tumor growth and tumor cell invasion by facilitating the engulfment of neighboring cells^183^.

**Fig. 11:**
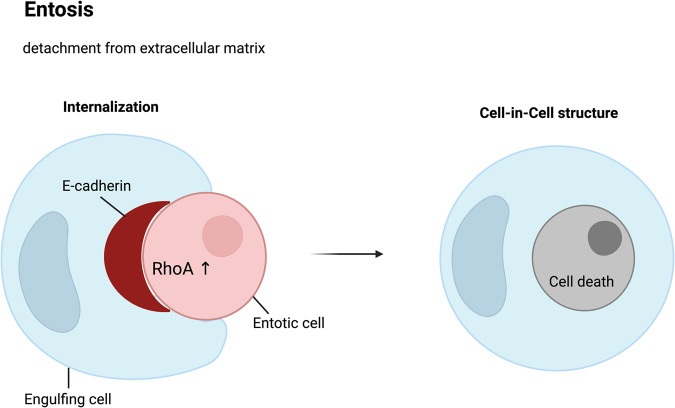
Cell-in-cell structures: a hallmark of entosis. Entosis is a biological process characterized by the internalization of one living cell into the cytoplasm of another. It is caused by adherent cell matrix separation, which results in the establishment of E-cadherin-mediated cell connections (shown in red) between the engulfing cell and the entotic cell. RhoA activity within the entotic cell causes actomyosin buildup at the cell cortex, resulting in the creation of cell-in-cell structures that mimic an active invasion-like process. Most internalized cells die as a result of entotic cell death, which is followed by lysosome fusion or apoptosis, especially when macroautophagy has been inhibited. However, certain entotic cells may divide within their hosts or even escape death.

Entosis is triggered when cells detach from the extracellular matrix (ECM) leading to the internalization of one cell by another cell. Entosis requires the activation of multiple molecular signaling pathways. One of the crucial pathways involved in entosis is the Rho/Rho-associated protein kinase (ROCK)/actomyosin pathway, which regulates actin and myosin II activities and is essential for cell engulfment^185^. The Rho/ROCK/actomyosin signaling pathway is involved in various processes, including cell migration, division, and shape changes^175^. This pathway involves the activation of the Rho family of small GTPases, which activate downstream effectors, such as ROCK^186^. In turn, ROCK activates myosin II, a motor protein that generates contractile forces by interacting with actin filaments^186^. During entosis, an invading (engulfed) entotic cell forms an actin-rich structure that protrudes into the cytoplasm of the engulfing cell^183^. Myosin II is recruited to this structure and contracts it, pulling the invading cell into the engulfing cell^187^. The internalized cells are subsequently degraded by lysosomes within the engulfing cell (Fig. 11)^182^.

Entosis is induced by various stimuli, such as nutrient starvation and ultraviolet radiation. Nutrient starvation, particularly glucose starvation, plays a pivotal role in inducing entosis by activating AMP-activated protein kinase (AMPK) in internal cells^188^, while ultraviolet radiation induces JNK and p38 stress-activated kinase signaling to activate entosis^189^. Entosis differs from anoikis, which is triggered by a lack of cell attachment to the ECM but not by matrix detachment^182^. Entosis shares more similarities to cell invasion than to a cell engulfment mechanism^182^. Entosis is also distinct from phagocytosis, which involves phagocyte engulfment of a dead or dying cell^190^.

Recent studies provided crucial insights into the mechanisms underlying entosis and its relevance in cancer development^191,192^. Specifically, Orai1, a Ca^2+^ channel protein, has emerged as a key player in entosis^193^. Orai1 plays a critical role in regulating intracellular Ca^2+^ levels during entosis, thereby influencing the activation of signaling pathway involved in cell engulfment and degradation. Dysregulation of Orai1-mediated Ca^2+^ signaling has been implicated in enhanced entosis, tumor growth, and invasion^194^. Although much is still unknown about the molecular mechanisms underlying entosis, this form of cell death has emerged as an important area of research with potential implications for both developmental biology and cancer therapeutics.

### Parthanatos

Parthanatos is a form of PCD that is mediated by the activation of poly ADP-ribose polymerase (PARP)^195^. The name is a combination of “PAR” and “*thanatos*” (the Greek word for death), reflecting the role of PAR-mediated cell death in various pathological conditions. It was first discovered by Karen Kate David in 2009^26^. This type of cell death was first identified in neurons and plays an important role in several neurodegenerative diseases, such as Alzheimer’s and Parkinson’s diseases^196^.

**Fig. 12:**
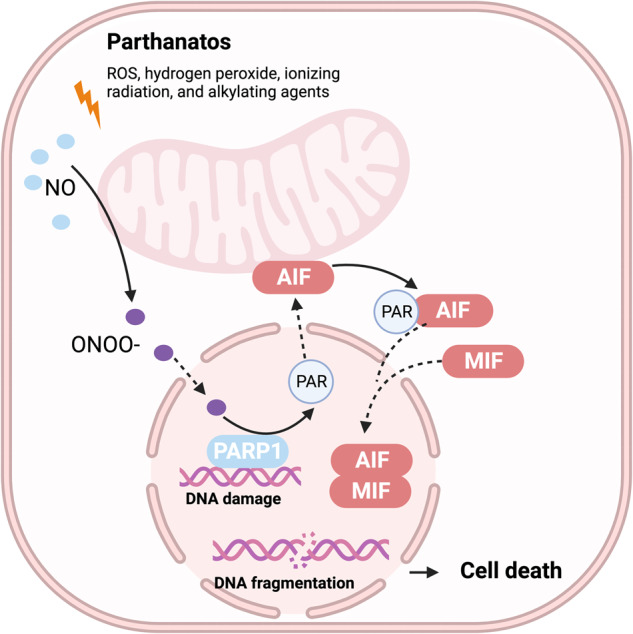
Mechanism underlying parthanatos. This diagram depicts the molecular processes underlying parthanatos. ROS, ischemia, alkylating chemicals, and radiation activate PARP-1 by activating NOS, resulting in the creation of excess NO and subsequent synthesis of peroxynitrite (ONOO^−^). Peroxynitrite activates PARP-1, resulting in the formation of copious amounts of PAR polymer in the nucleus. Certain poly(ADP)-ribosylated carrier proteins escape from the nucleus, prompting the outer mitochondrial membrane to release apoptosis-inducing factor (AIF). AIF then enters the cytoplasm and attaches to macrophage migration inhibitory factor (MIF). AIF and MIF enter the nucleus and cause widespread DNA degradation, ultimately resulting in cell death.

Parthanatos is triggered by various agents, such as ROS, hydrogen peroxide, ionizing radiation, and alkylating agents^195^. When DNA damage is mild, PARP-1 recruits DNA damage repair proteins to repair damaged DNA^197^. Severe DNA damage leads to PARP-1 overactivation and PAR polymer formation^198^. Accumulated PAR polymers bind to apoptosis-inducing factor (AIF) and mediate AIF release from mitochondria^199^. AIF interacts with MIF to form the AIF/MIF complex, which is translocated to the nucleus, causing DNA fragmentation and leading to parthanatos^200^ (Fig. 12).

Overall, parthanatos is a complex form of PCD that plays an important role in several disease processes. Further research is needed to fully understand the mechanisms underlying parthanatos and its potential therapeutic applications.

### Alkaliptosis

Alkaliptosis is a recently discovered form of regulated necrosis triggered by exposure to alkaline agents, such as ammonia, sodium hydroxide, or high-pH buffers^201^. Alkaliptosis was first discovered and named by Daolin Tang in 2018^201^. Alkaliptosis is a sequential molecular mechanism modulated by several factors.

**Fig. 13:**
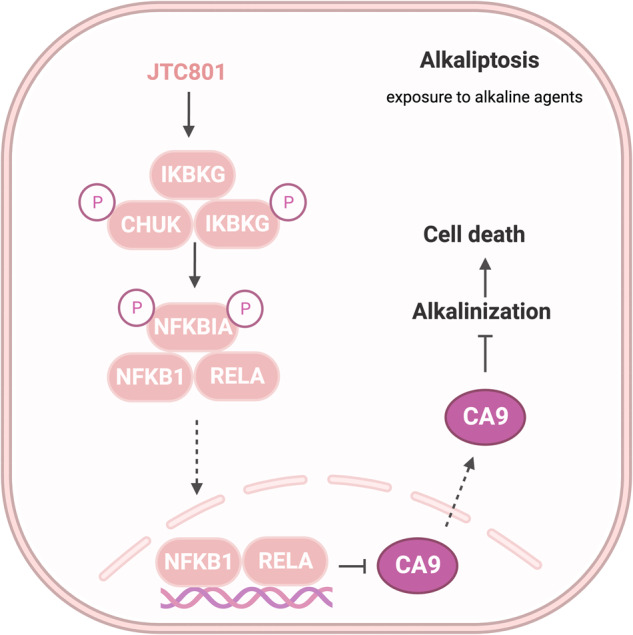
Molecular pathways in alkaliptosis. This figure illustrates the activation mechanism of alkaliptosis, which is characterized by intracellular alkalinization and subsequent cell death. JTC801 activates the IKK protein complex, which includes CHUK (IKKα), IKBKB (IKKβ), and IKBKG (IKKγ). Then, the IKK protein complex phosphorylates and degrades NFKBIA (IκBα), leading to the nuclear translocation of NFKB1 (p50) or RELA (p65), which regulate gene expression. Furthermore, NF-κB negatively regulates the expression of CA9, a member of the carbonic anhydrase family, to inhibit alkaliptosis.

Alkaliptosis can be activated by the upregulation of nuclear factor-kappa B (NF-κB) pathways and subsequent downregulation of carbonic anhydrase 9 (CA9) (Fig. 13)^29^. CA9 is a member of the carbonic anhydrase family that plays a role in regulating pH levels^29^. NF-κB negatively regulates CA9 activity, which in turn inhibits alkaliptosis^29^. Depletion of CA9 can restored the sensitivity of cancer cells that lack functional NF-κB to alkaliptosis^29,202^. Another study showed that ACSS2-mediated NF-κB activation promoted alkaliptosis in human pancreatic cancer cells^203^. ACSS2 has been found in the nucleus and cytoplasm and provides AcCoA, which is important for lipogenesis and histone acetylation^204^. ACSS2 plays a role in alkaliptosis by maintaining NF-κB activation and increasing the pH value via histone acetylation in human PDAC cells^203^.

Alkaliptosis is a promising strategy for cancer therapy because cancer cells have a profoundly unbalanced pH, and their proliferation, metastasis, and metabolic adaptation are determined by their pH sensitivity^205^. Drugs developed to target alkaliptosis may be a new approach to cancer treatment, especially for those resistant to conventional therapies^29^. However, further research is required to fully understand the mechanism of alkaliptosis and its potential for cancer therapy.

### Oxeiptosis

**Fig. 14:**
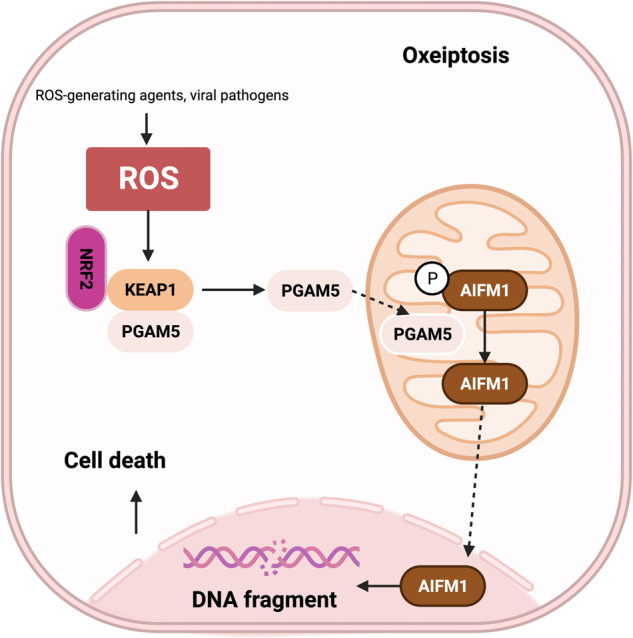
Mechanism underlying oxeiptosis. This figure illustrates the key features of oxeiptosis. Oxeiptosis is activated in response to oxidative stress induced by ROS or ROS-generating agents, such as viral pathogens. The KEAP1/PGAM5/AIFM1 signaling pathway plays a central role in oxeiptosis, in which AIFM1 is dephosphorylated under oxidative stress conditions via the regulatory action of PGAM5. Dephosphorylated AIFM1 is translocated from mitochondria to the nucleus, leading to chromatin condensation and DNA fragmentation, ultimately resulting in cell death.

The term “oxeiptosis” was coined by Holze et al. in 2018 to describe a caspase-independent, ROS-sensitive, and noninflammatory cell death pathway that protects against inflammation induced by ROS or ROS-generating agents, such as viral pathogens^30^. Oxeiptosis is characterized by the activation of the KEAP1/PGAM5/AIFM1 signaling pathway^206^. Under oxidative stress conditions, AIFM1 is dephosphorylated, and its activity is regulated by KEAP1 and PGAM5. Dephosphorylated AIFM1 is translocated from mitochondria to the nucleus, where it induces chromatin condensation and DNA fragmentation, leading to cell death (Fig. 14)^30^. Activation of the KEAP1/PGAM5/AIFM1 signaling pathway is a hallmark of apoptosis and differs from other cell death pathways, such as the apoptosis and necrosis pathways. The mechanisms underlying oxeiptosis are not yet fully understood, but it is thought to involve the activation of the nuclear factor erythroid 2-related factor 2 (Nrf2) pathway^207^.

Many conditions, such as allergies, autoimmune diseases, allograft rejection, cancer, and infection with pathogens, lead to ROS production, indicating that oxeiptosis may be triggered under various pathological conditions^208^. Notably, excessive or dysregulated oxeiptosis can lead to tissue damage and contribute to disease development. Understanding the mechanisms underlying oxeiptosis and its role in health and disease is an active area of research that may identify new therapeutic intervention targets.

### Erebosis

**Fig. 15:**
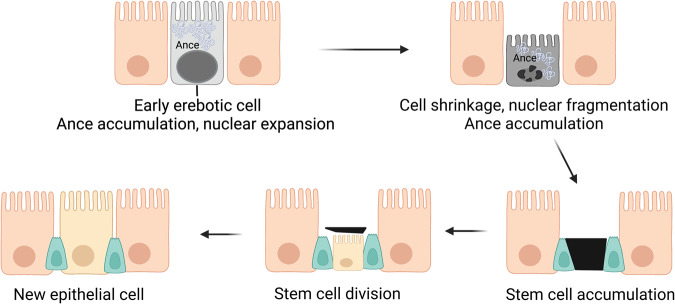
Structural characteristics of erebosis. This figure depicts the process of erebosis, a novel form of cell death observed during the natural turnover of enterocytes that constitute the gut epithelium. Nuclear expansion and accumulation of angiotensin-converting enzyme (ACE) are observed in the early stages of erebosis. Subsequently, cell shrinkage and nuclear fragmentation are observed. Late erebotic cells are surrounded by stem cells that eventually undergo division to generate new epithelial cells, contributing to the replenishment of the gut epithelium.

In 2022, Sa Kan Yoo et al. reported a novel form of cell death called erebosis during the natural turnover of gut enterocytes, which are the cells that make up the gut epithelium (Fig. 15)^32^. The term “erebosis” is derived from the Greek word “*erebos*,” meaning complete darkness^32^. Erebotic cells undergo membrane blebbing and actin cytoskeleton changes, eventually leading to cell disintegration^32^. This process is marked by the loss of cell adhesion, organelles, and fluorescence emitted from labeled proteins and the accumulation of angiotensin-converting enzyme (ACE)^209^. In contrast to cell undergoing apoptosis, necrosis, and autophagic cell death, cells undergoing erebosis do not exhibit distinguishing features. Notably, apoptosis inhibition does not affect apoptosis or gut cell turnover^209^. The authors suggested that erebosis may play a vital role in maintaining gut barrier function through the natural shedding of gut enterocytes^32^. In contrast, abnormal erebosis may contribute to the development of gastrointestinal conditions, such as inflammatory bowel disease^32^. Although this phenomenon has only been discovered in *Drosophila*, further research is required to determine whether it also occurs in other organisms, including humans. Such investigations may provide insights into the evolutionary origins and physiological significance of erebosis and enhance our understanding of gut physiology and related disorders.

## The complexity underlying cell death

### The complexity of cell death classification

The categorization system for cell death is intrinsically complexity. Initially, cell death was classified primarily based on morphological characteristics, as described below^2^. Type 1 cell death, known as apoptosis, is characterized by cytoplasm shrinkage, chromatin condensation, membrane blebbing, and the formation of apoptotic bodies, which are cleared through phagocytosis and lysosomal degradation by the surrounding cells^210^. Type 2 cell death is characterized by intense autophagy or cytoplasmic vacuolization, leading to phagocytosis by neighboring cells and degradation via lysosomes^210^. Type 3 cell death, or necrosis, results in cell death without triggering phagocytosis or lysosomal degradation and is not characterized by any of the features used to identify type 1 and 2 cell death modalities^210^. As more forms of cell death were identified, type 4 cell death modalities were classified, and these types of cell death includes those that cannot be classified into one of the three previously defined categories^211^. However, the current classification system of cell death, based on morphological changes, is limited because newly discovered forms of cell death cannot be integrated into it. Furthermore, the existing classification system does not account for the increasingly recognized importance of molecular pathways, specifically their greater importance than morphological changes, in identifying forms of cell death^210^. Thus, there is a need for more comprehensive guidelines that are based on genetic, biochemical, and functional criteria.

**Fig. 16:**
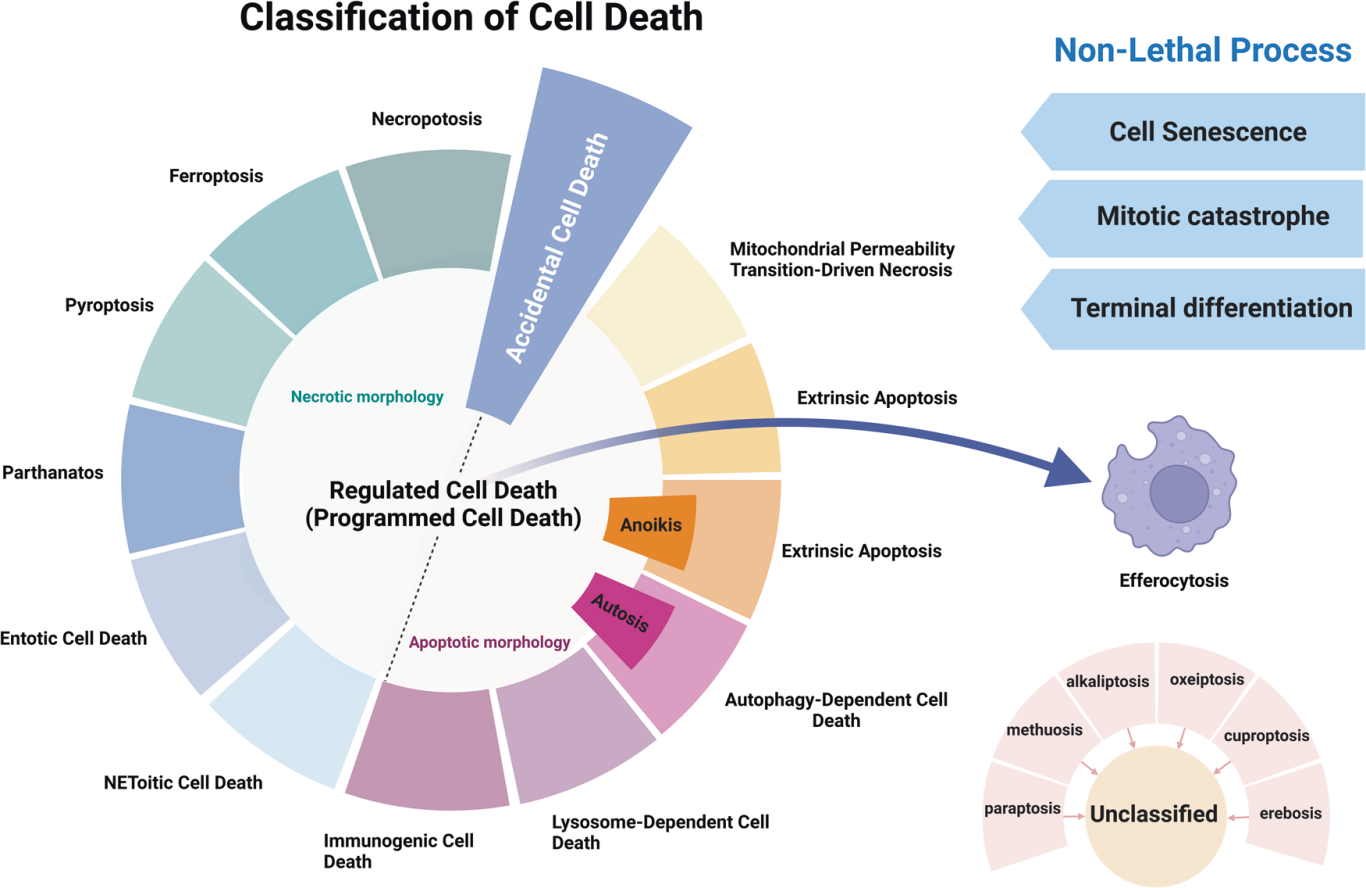
Classification of cell death. This figure illustrates the classification of the different forms of cell death and nonlethal processes based on their underlying mechanisms and morphological features. This figure was generated according to the 2018 guidelines for the classification of cell death issued by the Nomenclature Committee on Cell Death (NCCD).

To address this need, the NCCD issued guidelines on the “classification of cell death” in 2005^212^, 2009^213^, and 2018^2^. The 2018 classification system aimed to establish a more comprehensive system based on genetic, biochemical, and functional criteria, not merely morphological features (Fig. 16)^2^. However, this system also has several limitations. First, certain forms of cell death, namely, paraptosis, methuosis, alkaliptosis, oxeiptosis, cuproptosis, and erebosis, were not classified. Second, although pyroptosis, NETosis, and necroptosis are categorized as different types of cell death^67^, these modalities all involve immunogenic cell death^98^. Finally, the current classification system may not account for the potential interplay and crosstalk among different forms of cell death because cell death processes are mediated via a complex network of interactions contributing to cellular processes that may not be fully reflected in the classification system.

### Complexity of the interconnectedness among different types of cell death

**Fig. 17:**
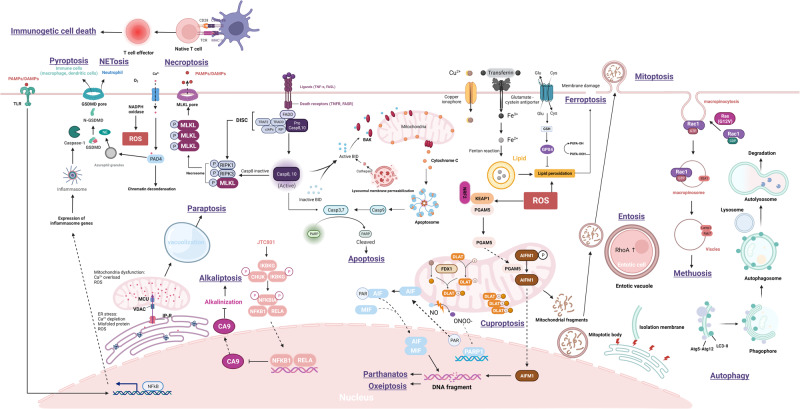
Complexity of cell death. This figure illustrates the complex and interconnected nature of cell death pathways. The figure shows the mechanisms by which different types of cell death pathways interact and influence each other and the ways in which they can be regulated by various signaling pathways and environmental factors.

The interconnectedness among the various forms of cell death is an important factor that contributes to cell death complexity (Fig. 17). Necroptosis, a regulated form of necrosis, shares similarities with necrosis and apoptosis^131,133^. Autophagy is required for both autosis and autophagic cell death programs, which are triggered via different molecular pathways^53,75^. Similarly, cells undergoing parthanatos display morphological and cytological characteristics of both apoptosis and necrosis^26^. Recent studies have suggested that autophagy and ferroptosis pathways interact in a complex manner. Autophagy has been suggested to regulate ferroptosis by eliminating damaged mitochondria and peroxidized lipids^214^. However, excessive or prolonged autophagy can lead to ferritin degradation, which can trigger ferroptosis^215^.

Cell death is influenced by various factors, including cellular organelles and environmental conditions. Mitochondria play crucial roles in different types of cell death, including apoptosis, NETosis, paraptosis, parthanatos, and oxeiptosis^216^. For example, apoptosis is triggered by mitochondrial outer membrane permeabilization (MOMP), which leads to the release of cytochrome c and activation of the caspase cascade^216,217^. In cells undergoing NETosis, mitochondria produce mitochondrial ROS (mtROS), whereas in cells undergoing paraptosis, Ca^2+^ released from the ER causes mitochondrial Ca^2+^ overload and mitochondrial dilation^218,219^. Both parthanatos and oxeiptosis involve mitochondrial release of AIF, which is translocated from the mitochondria to the nucleus, resulting in cell death^30,195^.

Lysosomes play several roles in cell death. During lysosomal cell death, lysosomal membrane permeabilization results in the release of cathepsins, which activate apoptotic pathways^83^. During necrosis, lysosomal membrane permeabilization causes the release of lysosomal hydrolases and ROS, which cause cell damage and inflammation [183]. In cells undergoing autophagic death, lysosomes fuse with autophagosomes to degrade cellular components, leading to cell death^220^. Lysosomal exocytosis has been implicated in the induction of pyroptosis, a type of inflammatory cell death^221^. In addition, endothelial cells undergo lysosomal degradation^182^.

Similar to lysosomal factors, ROS trigger cell death in various ways. For example, ROS play a crucial role in activating NADPH oxidase, which is required for the degradation of azurophilic granules that trigger NETosis^116^. Moreover, ROS can cause lipid peroxidation, leading to ferroptosis^27^. Additionally, ROS can cause ER stress by inducing the accumulation of unfolded proteins, triggering paraptosis^222^. ROS production is also the primary cause of oxeiptosis initiation^30^. Furthermore, a rapid increase in the cytosolic pH vale can induce both lysosomal cell death and alkaliptosis^17,29^. Some types of cell death, such as paraptosis and methuosis, are caused by the formation of large vacuoles, highlighting the importance of organelle dysfunction in regulating cell death^223^.

Overall, the interconnectedness among the different types of cell death and their regulation via diverse signaling pathways and environmental factors highlight the complexity of cell death. Understanding the interplay among different signaling pathways and the impact of the cell context on the cell death modality is crucial for developing new therapeutic strategies that target cell death pathways for the treatment of various diseases. Further research is needed to fully characterize and differentiate among the various forms of cell death and their roles in health and disease.

### PANoptosis: an emerging and complex form of cell death

**Fig. 18:**
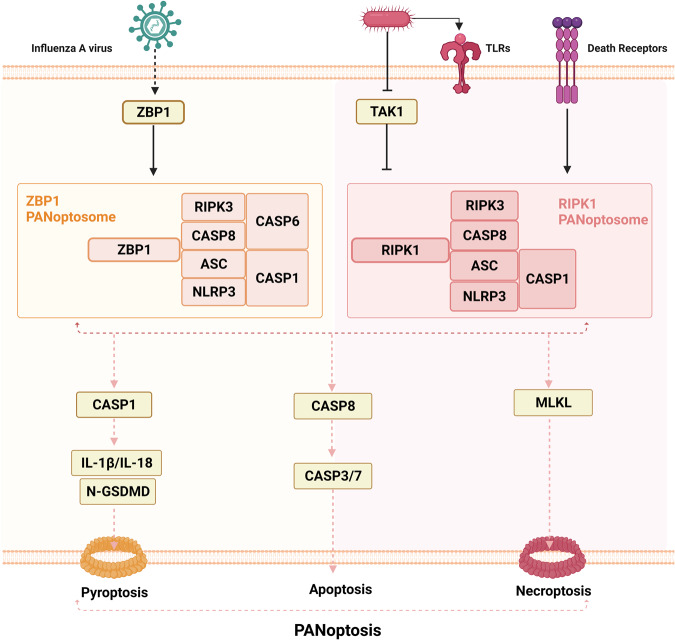
Overview of PANoptosis. PANoptosis is triggered by the formation of a protein complex called the PANoptosome, which includes several protein domains, namely, RIPK1, RIPK3, caspase-8, NLRP3, and ASC. This complex activates multiple types of cell death, including pyroptosis, apoptosis, and necroptosis, resulting in an inflammatory cell death response. During influenza A virus (IAV) infection, Z-DNA-binding protein (ZBP1) recognizes viral ribonucleoproteins and induces the formation of the ZBP1-dependent PANoptosome. TGF-β-activated kinase 1 (TAK1) is a crucial regulator of PANoptosis that negatively controls this process; however, bacterial infections can interrupt its suppression. Inhibition of TAK1 and activation of signaling through TLRs or death receptors promotes the formation of RIPK1-dependent PANoptosomes. During PANoptosis, the activation of caspase-1 or caspase-8 leads to the cleavage and activation of downstream effector proteins, such as gasdermin D and RIPK3, which drive pyroptosis and necroptosis, respectively. Activated caspase-8 subsequently cleaves and activates caspase-3, resulting in cell apoptosis.

In addition to the different types of cell death that share the same molecular pathways, it has recently been shown that different types of cell death are mediated simultaneously in a single cell. This phenomenon was first described by Kanneganti et al. in 2016 when they studied inflammasome activation by influenza virus^224^ and named it PANoptosis in 2019 (Fig. 18)^225^. PANoptosis is triggered by the formation of a protein complex called the PANoptosome, which is composed of several proteins, including RIPK1, RIPK3, caspase-8, NLRP3, and ASC^226^. This complex activates various cell death pathways, including pyroptosis, apoptosis, and necroptosis, resulting in an inflammatory cell death response^226^.

During influenza A virus (IAV) infection, Z-DNA-binding protein (ZBP1) plays a critical role in activating PANoptosis by recognizing viral ribonucleoproteins and inducing the formation of the ZBP1-dependent PANoptosome^224,227^. This complex consists of ZBP1 (the sensor), RIPK3, RIPK1, NLRP3, ASC, caspase-1, caspase-8, and scaffold caspase-6^228^. TGF-β-activated kinase 1 (TAK1) is a crucial regulator of PANoptosis that negatively controls its initiation. Notably, bacterial infections can interrupt PANoptosis suppression via the action of the Yersinia T3SS effector YopJ, leading to PANoptosome formation^225^. When TAK1 was inhibited and signaling through TLRs or death receptors was activated, RIPK1-dependent PANoptosomes were formed^229^.

During PANoptosis, the activation of caspase-1 or caspase-8 leads to the cleavage and activation of downstream effector proteins, such as gasdermin D and RIPK3, which drive pyroptosis and necroptosis, respectively^226^. In addition, activated caspase-8 can cleave and activate caspase-3, leading to apoptosis^230^. The pathophysiological functions and importance of phagocytosis in relation to viral infections have been extensively studied during the COVID-19 pandemic; however, clear evidence of a phagocytic protein complex has yet to be established. Further research is required to identify this complex or eliminate the possibility that a complex is formed.

## Conclusion and future perspectives

In the preceding sections, we discussed the many types of cell death and history of their discovery. Understanding the diverse and complex processes underlying cell death is crucial for understanding diseases and may be beneficial for the development of new therapies. The classification of cell death based on morphological features is limited because it cannot accommodate newly discovered forms of cell death and may not reflect the underlying molecular pathways that determine a form of cell death. Therefore, recent guidelines proposed by the NCCD aim to establish a more comprehensive classification scheme based on genetic, biochemical, and functional criteria^2^. Moreover, several new forms of cell death have been discovered that are not included in the latest NCCD classification, and some scholars have disputed the usefulness of this classification. We hope that the next NCCD consensus will produce a new cell death classification system that addresses the aforementioned issues.

Researchers have made significant strides in characterizing and distinguishing various forms of cell death, thereby advancing our understanding of the roles of these modalities in health and disease. The complex mechanisms underlying cell death are underscored by the intricate interconnections among different types of cell death and the regulation of these mechanism through diverse signaling pathways and environmental factors. In addition, the importance of crosstalk among signaling pathways and the influence of the cellular context on cell death outcomes has become increasingly evident. These findings pave the way for the development of novel therapeutic strategies targeting cell death pathways for the treatment of diverse diseases. Further research is crucial to characterize and differentiate various forms of cell death, to gain a better understanding of their roles in disease progression, and to develop targeted therapeutic strategies (Table 2). Ultimately, a comprehensive understanding of the multifaceted nature of cell death will be indispensable for the development of innovative and more efficacious treatments for a broad spectrum of diseases.

**Table 2:** List of cell death-related diseases.

Type	Disease	Reference
Necrosis	Inflammation and damage to surrounding tissues	^37^
Apoptosis (Deficient)	Cancer, autoimmune disorders, and viral infections	^231^
Apoptosis (Excessive)	Ischemic heart disease, stroke, neurodegenerative diseases, sepsis, and multiple organ dysfunction syndrome	^231^
Autophagic cell death	Cancer, neurodegeneration, ischemic injury, and heart disease	^70–74^
Autosis	Severe liver disease	^82^
Entosis	Cancer	^191,192^
Methuosis	Cancer	^180,181^
Mitoptosis	Mitochondrion-associated human diseases	^232^
Pyroptosis	Infectious diseases, autoimmune disorders, cancer, and neurodegenerative diseases	^112,113^
NETosis	Inflammatory and autoimmune disease	^11,12^
Necroptosis	Neurodegenerative diseases, viral infections, ischemic injury, and cancer	^141–144^
Parthanatos	Smoke-related lung diseases, macular degeneration, Parkinson’s disease, and oxidative stress-related hearing disorders	^233–236^
Ferroptosis	Acute kidney injury, cancer, cardiovascular disorders, neurodegenerative conditions, and hepatic diseases	^237^
Cuproptosis	Wilson’s disease	^154^
Oxeiptosis	Allergies, autoimmunity, allograft rejection, cancer, and infection with pathogens	^208^

As we gain a better understanding of new types of cell death and their complexities, the study of cell death is becoming increasingly difficult. In particular, different processes of cell death are linked by molecular mechanisms and, in some cases, are potentially coactivated. Because of these connections, the specificities of molecular markers used to distinguish among different types of cell death are becoming increasingly ambiguous. Furthermore, whether the agents used to inhibit specific cell death are sufficiently specific is an ongoing concern. Developing new inhibitors with greater specificity or modulating key genes may solve the problems associated with lack of specificity among inhibitors. However, the emergence of complex forms of cell death suggests that the inhibition of only one type of cell death may not be sufficient to achieve therapeutic results. Therefore, future studies on cell death may require an integrated view of different types of cell death.

A scientist studying death of a cell can be compared with a forensic physician investigating a crime scene. However, in contrast to forensic physicians who focus on identifying the cause of a murder, scientists focused on cell death are primarily interested in understanding the mechanisms and types of PCD, that is, cell suicide. From a forensic standpoint, some may question the need for such a detailed classification and identification of cell death. However, understanding the processes and types of cell death is important to fully comprehend the manner in which cells resolve internal disharmony and maintain balance. Therefore, scientists interested only in cell suicide will play an increasingly important role in the cellular universe, similar to forensic scientists investigating crime scenes in the macroscopic world.
