# Author Correction: Diversity and complexity of cell death: a historical review

**DOI:** 10.1038/s12276-023-01107-9

**Published:** 2023-09-20

**Authors:** Wonyoung Park, Shibo Wei, Bo-Sung Kim, Bosung Kim, Sung-Jin Bae, Young Chan Chae, Dongryeol Ryu, Ki-Tae Ha

**Affiliations:** 1 Department of Korean Medical Science, School of Korean Medicine, Pusan National University, Yangsan, Gyeongsangnam-do 50612 Republic of Korea; 2 Korean Medical Research Center for Healthy Aging, Pusan National University, Yangsan, Gyeongsangnam-do 50612 Republic of Korea; 3 Department of Precision Medicine, School of Medicine, Sungkyunkwan University School of Medicine, Suwon, Gyeonggi-do 16419 Republic of Korea; 4 Department of Molecular Biology and Immunology, Kosin University College of Medicine, Busan, 49267 Republic of Korea; 5 Department of Biological Sciences, UNIST, Ulsan, 44919 Republic of Korea; 6 Department of Biomedical Science and Engineering, Gwangju Institute of Science and Technology, Gwangju, 61005 Republic of Korea

**Keywords:** Apoptosis, Autophagy, Entosis, Necroptosis

Correction to: *Experimental & Molecular Medicine* 10.1038/s12276-023-01078-x, published online 23 August 2023

After online publication of this article, the authors noticed an error in the “cuproptosis” section.

The correct statement of this article should have read as below.

“This was first described and the term was initially defined by Tsvetkov, P. et al. in 2019 (Nat Chem Biol 15, 681–689, 2019).”

The authors apologize for any inconvenience caused. The original article has been corrected.

